# CHIP amplifies the risk of lymphoid malignancies in individuals with monoclonal B-cell lymphocytosis (MBL)

**DOI:** 10.1038/s41408-025-01385-8

**Published:** 2025-11-06

**Authors:** Nicholas J. Boddicker, Tait D. Shanafelt, Sameer A. Parikh, Kari G. Rabe, Rosalie Griffin, Aaron D. Norman, Yuan Yao, Shulan Tian, Tao Ma, Daniel R. O’Brien, Bryan A. Vallejo, Mingma S. Hoel, Stacey J. Lehman, Janet E. Olson, Paul J. Hampel, Esteban Braggio, Mrinal M. Patnaik, James R. Cerhan, Celine M. Vachon, Curtis A. Hanson, Susan L. Slager

**Affiliations:** 1 Division of Computational Biology, Mayo Clinic, Rochester, MN USA; 2 Department of Medicine, Division of Hematology, Stanford University, Stanford, CA USA; 3 Division of Hematology, Mayo Clinic, Rochester, MN USA; 4 Division of Clinical Trials and Biostatistics, Mayo Clinic, Rochester, MN USA; 5 Division of Epidemiology, Mayo Clinic, Rochester, MN USA; 6 Department of Cancer Biology, Mayo Clinic, Phoenix, AZ USA; 7 Department of Laboratory Medicine and Pathology, Mayo Clinic, Rochester, MN USA

**Keywords:** Cancer epidemiology, Lymphoma

## Abstract

Monoclonal B-cell lymphocytosis (MBL) and clonal hematopoiesis of indeterminate potential (CHIP) are prevalent clonal precursors associated with increased risk of lymphoid malignancies. However, the relationship between MBL and CHIP and their combined impact on lymphoid malignancy risk remains poorly understood. We screened participants from the Mayo Clinic Biobank to identify MBL using eight-color flow cytometry; CHIP was detected using whole-exome sequencing of whole-blood DNA in 291 genes related to myeloid or lymphoid malignancies. Incident myeloid or lymphoid hematological malignancies were identified using ICD codes and confirmed via medical record review. Logistic regression was used to estimate odds ratios (OR) and 95% confidence intervals (CI). Cox regression was used to estimate hazard ratios (HR). Analyses were adjusted for age and sex. In 10,067 participants, 15% had MBL, and 9% had CHIP. No evidence of an association between MBL and CHIP (OR = 1.00; 95% CI: 0.82–1.20) was observed. With a median follow-up of 5.4 years, 138 participants developed hematological malignancies (94 lymphoid). MBL (HR = 3.48; 95% CI: 2.27–5.34; *P* < 0.001) and CHIP (HR = 1.89; 95% CI: 1.10–3.27; *P* = 0.022) were each associated with incident lymphoid malignancy. Compared to individuals with no precursors, the combined presence of MBL and CHIP significantly amplified lymphoma risk (HR = 7.18; 95% CI: 3.33–15.47; *P* < 0.001), more than doubling the risk among individuals with MBL alone (HR = 3.30; 95% CI: 2.06–5.30; *P* < 0.001). In contrast, the risk associated with CHIP alone was attenuated and no longer statistically significant (HR = 1.63; 95% CI: 0.77–3.47; *P* = 0.20). MBL and CHIP are independent hematological precursor conditions. While their combined presence amplifies the risk of lymphoid malignancy, CHIP alone may not be a strong independent risk factor.

## Introduction

Monoclonal B-cell lymphocytosis (MBL) and clonal hematopoiesis of indeterminate potential (CHIP) are asymptomatic clonal hematological conditions detectable in the blood of otherwise healthy individuals. MBL is a precursor condition to chronic lymphocytic leukemia (CLL) [1–3] and is characterized by an absolute clonal B-cell count of <5 × 10^9^/L without lymphadenopathy, organomegaly, or cytopenia [4–6]. It is classified according to cell-surface immunophenotype (CLL or non-CLL phenotype) and clone size: high-count MBL (HC-MBL, clonal B-cell between 0.5 and 5 × 10^9^/L), and low-count MBL (LC-MBL; clonal B-cell <0.5 × 10^9^/L) [6]. In the general population, MBL affects 5–10% of adults over the age of 40 years, with prevalence increasing with age [7, 8]. Using a subset of participants from the current study, we reported that individuals with CLL-type MBL have 6.8-fold (95% CI: 2.6–18.1) increased risk of incident lymphoid malignancies [9].

CHIP is characterized by clonal populations of blood cells with acquired somatic mutations in genes primarily linked to myeloid malignancies among individuals without a hematological malignancy [10–13]. Its prevalence in the general population is ~10% and increases with age [14]. Studies have shown that individuals with CHIP have an increased risk of myeloid malignancies [10, 11, 13, 15–18]. Recently, the definition of CHIP has expanded to include mutations in 235 genes related to lymphoid malignancies, and individuals with CHIP mutations in these lymphoma-related genes were reported to have a 4.2-fold (95% CI: 2.7–6.7) increased risk of incident lymphoid malignancies and a 20-fold (95% CI: 11.1–38.0) risk for incident CLL, specifically [17].

The prior studies examining the association between CHIP and risk of lymphoid malignancy did not account for MBL status, raising the possibility that what was being classified as CHIP was actually detecting genetic abnormalities in an established MBL clone. As a result, the relationship between MBL and CHIP and their combined impact on hematological malignancy risk remains poorly understood. To address this, we analyzed data from our cohort of over 10,000 individuals screened for both CHIP and MBL.

## Methods

### Ethics approval and consent to participate

This study was reviewed and approved by the institutional review board of Mayo Clinic and Olmsted Medical Center (IRB# 15-004986). All methods were performed in accordance with the relevant guidelines and regulations. Written informed consent was obtained from all participants.

### Study population

Study participants were from the Mayo Clinic Biobank, a large-scale biorepository of individuals recruited from primary care clinics between 2009 and 2016 [19]. Details on the MBL screening cohort have been previously published [9, 20–23]. In brief, a random sample of 10% of the participants was selected to have peripheral blood mononuclear cells (PBMCs) banked, and thus were available to screen for MBL. From this random sample, we excluded individuals with a prior history of hematological cancer and those <40 years of age at the time of their phlebotomy, resulting in our MBL Screening Cohort 1. In Cohort 1, the blood sample collected at the time of enrollment was used to screen for both MBL and CHIP. Starting in 2017, we assembled a second cohort (Cohort 2) for MBL screening derived from the 90% of individuals participating in the Mayo Clinic Biobank who were not randomized to have stored PBMCs. New blood samples were collected from Cohort 2 participants who resided in the 27 counties surrounding Mayo Clinic, Rochester, MN, were 40 years or older, and had no prior history of hematologic malignancy. Note that CHIP screening in Cohort 2 was conducted on the original whole blood samples collected at the time of consent into the Mayo Clinic Biobank (i.e., the earlier of the two samples) and not the PBMC samples used to screen for MBL.

### MBL screening

Our MBL screening strategy has been detailed previously (Supplementary Methods) [9, 20–23]. Across the two cohorts, frozen PBMCs were screened for MBL using eight-color flow cytometry with the capacity to detect clonal B-cell events with a sensitivity of 0.005% (1/20,000 events). MBL immunophenotype was categorized, and individuals whose major clone immunophenotype was non-CLL-type MBL were excluded from the present study. As previously validated, individuals with MBL were classified as LC-MBL if <85% of B-cells were clonal B-cells and HC-MBL if ≥85% of B-cells were clonal B-cells [2, 24].

### CHIP screening

CHIP screening was performed on DNA from whole blood using whole exome sequencing with a mean coverage depth of 48X (Supplementary Methods). CHIP variants included all protein-truncating and missense variants in known hotspot regions in the 291 genes (Supplementary Table 1) related to myeloid (*N* = 56) or lymphoid malignancy (*N* = 235), per Niroula et al. [17]. Among these 291 genes, we note 43 genes have been previously associated with CLL (Supplementary Table 1) [25–28]. We assessed the sensitivity of WES for detecting CHIP by comparing it to targeted sequencing with >1000x coverage in 42 CHIP genes in 1023 individuals (Supplementary Methods). While the concordance of WES with the targeted sequencing was high for CHIP clones with variant allele fraction (VAF) > 10%, WES missed small CHIP clones identified in targeted sequencing and therefore below WES detection sensitivity.

### Incident hematologic malignancies ascertainment

Incident hematological malignancies were identified using ICD codes and confirmed through medical record review. Diagnoses were obtained from individuals seen at Mayo Clinic, Rochester, MN, or individuals included in the Rochester Epidemiology Project (REP), a population-based medical records-linkage system of complete medical records from all medical facilities in the 27 counties surrounding Mayo Clinic, Rochester, MN [29, 30]. The reviewers confirming diagnoses were blinded to MBL and CHIP status of the individuals.

### Statistical analysis

Cohorts 1 and 2 were combined for analyses. Comparisons of demographic factors and precursor status were performed using rank sum tests, Chi-square tests, or Fisher’s Exact tests, where appropriate. We used logistic regression to evaluate the association between CHIP and MBL status, adjusting for sex and age at the time of MBL screening to estimate odds ratios (ORs) and 95% confidence intervals (CIs). Cumulative incidence of malignancy was computed using the Aalen-Johansen method, where death was considered a competing event. Inverse probability weights were applied to the cohorts so that the age and sex distributions were similar between the groups. Individuals were followed from the date of sample collection used for MBL screening to the earliest date of incident malignancy, withdrawal, death, last medical visit, or study end (March 15, 2025). We used Cox regression to evaluate the association of the precursor conditions with incident malignancies, adjusting for age and sex; the cohort was included as a strata in the model. Hazard ratios (HR) and 95% CI were estimated. We next evaluated the co-occurrence of both precursors with incident malignancies (either lymphoid or myeloid) by modeling those individuals with (1) both precursors, (2) those with MBL alone, (3) those with CHIP alone, and (4) those without MBL or CHIP (reference group). Models were adjusted for age and sex; the cohort was included as a strata in the model. The Cox regression proportional hazards assumption was tested using the R functions coxph and cox.zph, and the models met the assumptions. We used the threshold of *P* < 0.05 to determine statistical significance.

## Results

### Patient characteristics

**Fig. 1:**
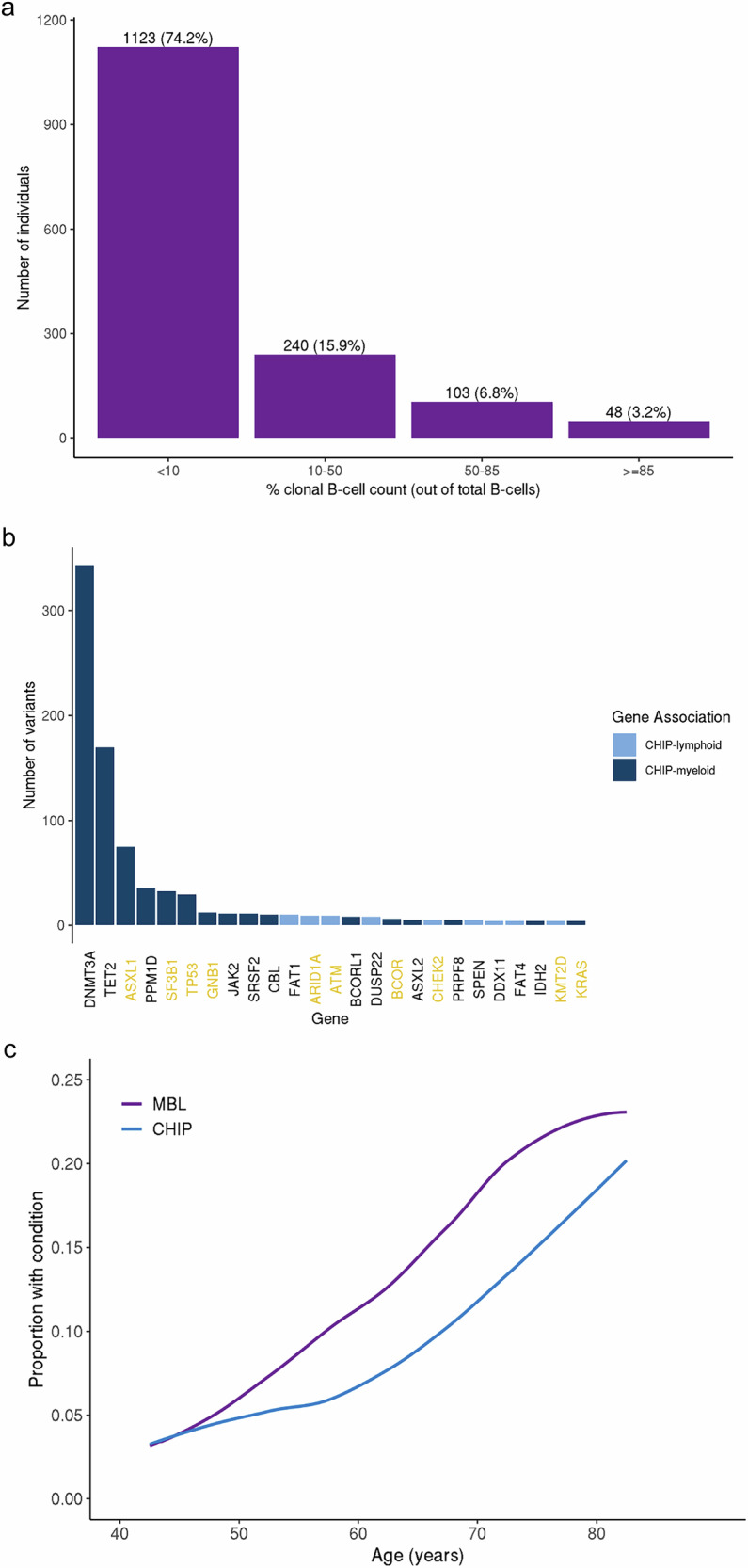
Individual characteristics of monoclonal B-cell lymphocytosis (MBL) or clonal hematopoiesis of indeterminate potential (CHIP). **a** Distribution of percent clonal B-cell count out of total B-cell count in 1514 individuals with MBL. **b** Distribution of CHIP variants by gene (top 25 genes shown). Bars are colored based on the gene association with either myeloid (*N* = 56 genes) or lymphoid (*N* = 235 genes) malignancy. Gene names are colored (gold) to reflect the subset of CHIP genes associated with chronic lymphocytic leukemia (CLL, *N* = 43 genes). A total of 969 variants were identified in 867 individuals. **c** Prevalence of precursor condition across age.

This study included 10,067 individuals with both MBL and CHIP screening results (Supplementary Table 2). Median age at the time of MBL screening was 66 years, 38.1% were male, and 1514 (15.0%) individuals screened positive for CLL-phenotype MBL (subsequently referred to as MBL). The distribution of the percent clonal B-cell count out of total B-cell count among those individuals with MBL is shown in Fig. 1a, with 96.8% (*N* = 1466) LC-MBL and 3.2% (*N* = 48) HC-MBL. We identified 867 (8.6%) individuals with CHIP variants, with the top genes (*DNMT3A, TET2*, and *ASXL1*) accounting for 60.7% of the CHIP variants (Fig. 1b, Supplementary Table 3). Among the top 15 genes with CHIP variants, the median variant allele frequency (VAF) was highest for *SRSF2* (median VAF = 0.22, Supplementary Fig. 1). Notably, CHIP variants in genes also related to CLL (including *ASXL1*, *SF3B1*, and *TP53*, Fig. 1b, Supplementary Table 3) accounted for 22% of the CHIP variants. As expected, both MBL and CHIP were associated with aging (Fig. 1c, Supplementary Fig. 2).

### Relationship between MBL and CHIP

**Fig. 2:**
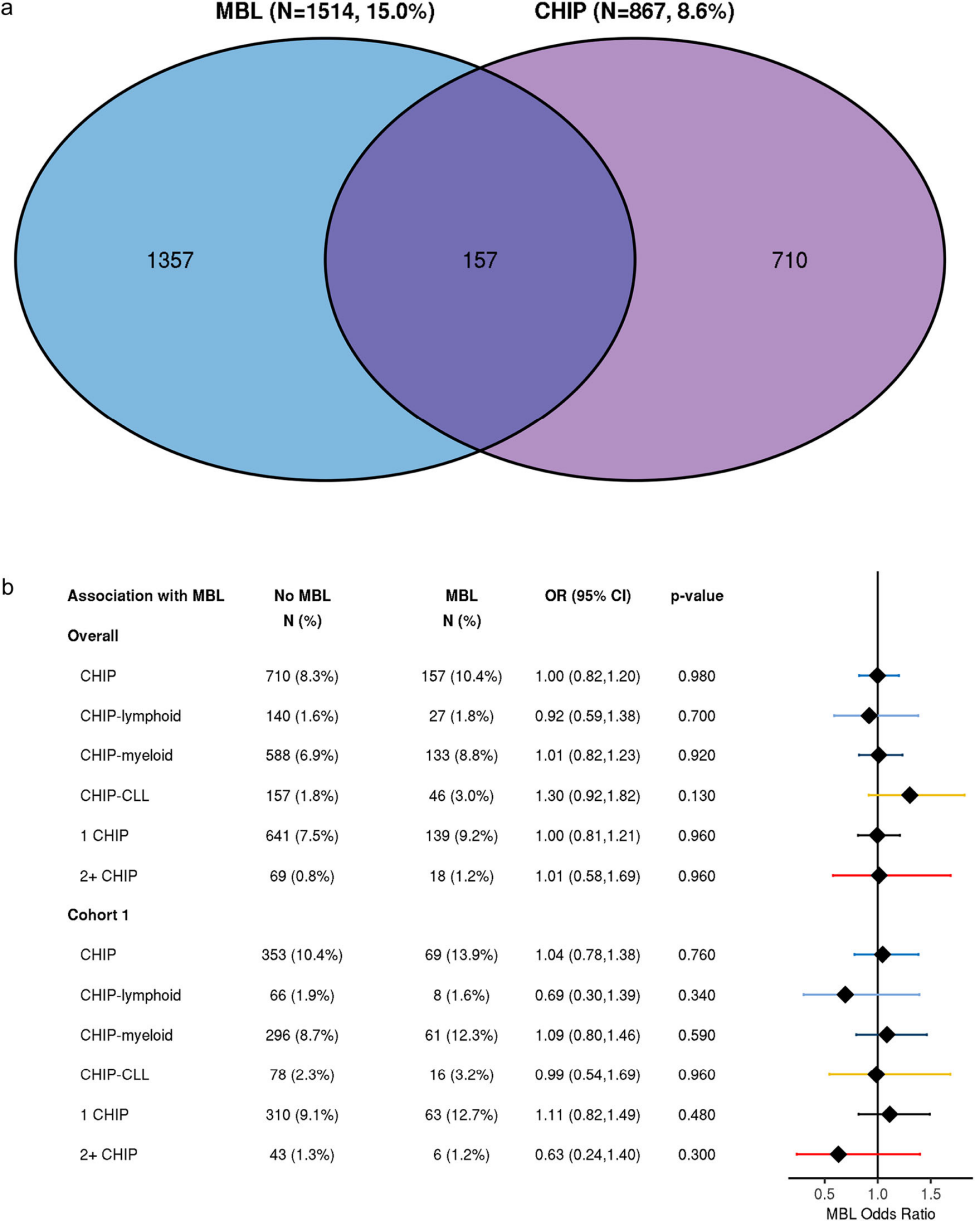
Relationship between monoclonal B-cell lymphocytosis (MBL) and clonal hematopoiesis of indeterminate potential (CHIP). CHIP is defined as variants in genes associated with either myeloid (*N* = 56 genes) or lymphoid (*N* = 235 genes). **a** Venn diagram of the co-occurrence of MBL and CHIP. **b** Association between MBL and CHIP overall, and by Cohort 1, in which the same blood sample was used for MBL and CHIP screening. CHIP-lymphoid is defined as variants present in 235 genes associated with lymphoid malignancy. CHIP-myeloid is defined as variants present in 56 genes associated with myeloid malignancy. CHIP-CLL is defined as variants in a subset of 43 genes associated with chronic lymphocytic leukemia (CLL). CHIP-CLL is not independent from CHIP-lymphoid and CHIP-myeloid. Individuals with 2 or more CHIP variants are defined as 2 + CHIP.

We investigated the association between CHIP and MBL. While 2224 (22.1%) individuals screened positive for at least one precursor, only 157 (1.6%) had both (Fig. 2a). The frequency of CHIP was 8.3% (*N* = 710) in individuals without MBL and 10.4% (*N* = 157) located across 119 genes in those with MBL. The percent clonal B-cell count out of total B-cell count was similar between those with and without CHIP (*P* = 0.38). The frequency of MBL was 14.8% (*N* = 1357) in those individuals without CHIP and 18.1% (*N* = 157) in those with CHIP. Using multivariable logistic regression adjusting for age and sex, no evidence of an association between CHIP and MBL was identified (OR = 1.00, 95% CI: 0.82–1.20, *P* = 0.98, Fig. 2b). This non-significant association between CHIP and MBL held when we stratified participants based on CHIP gene type (lymphoid- or myeloid-related) or whether analysis was restricted to Cohort 1, in which CHIP and MBL were measured on the same blood draw (Fig. 2b). Although an elevated but non-significant association between MBL and CHIP in CLL-related genes was observed (OR = 1.30, 95% CI: 0.92–1.82, *P* = 0.13), the effect attenuated (OR = 0.99, 95% CI: 0.54–1.69, Fig. 2b) when restricting to individuals in Cohort 1.

### Association between precursor conditions and lymphoid malignancies

We followed individuals from the time of MBL screening for the development of incident lymphoid malignancies. Median follow-up was 5.4 years (range 0–15.7); 94 individuals developed incident lymphoid malignancies (Supplementary Table 4). The most common lymphoid subtypes included multiple myeloma (*N* = 17), CLL (*N* = 16), and diffuse large B-cell lymphoma (*N* = 16).

Using multivariate Cox regression adjusting for age and sex, we found that individuals with MBL had a 3.48-fold (95% CI: 2.27–5.34, *P* < 0.001, Table 1) increased risk of incident lymphoid malignancies compared to individuals without MBL. Individuals with LC-MBL had a 2.62-fold increased risk (95% CI: 1.63–4.20, *P* < 0.001, Table 1). When the diagnosis of CLL was excluded from this analysis, individuals with MBL had a 2.29-fold increased risk (95% CI: 1.39–3.75, *P* = 0.001) of incident lymphoid malignancy. When including only the diagnosis of CLL, individuals with MBL had a 26.86-fold increased risk (95% CI: 7.40–97.50, *P* < 0.001). Individuals with CHIP in any of the 291 genes evaluated had a 1.89-fold (95% CI: 1.10–3.27, *P* = 0.022, Table 1) increased risk of lymphoid malignancy compared to individuals without CHIP. When CHIP variants were limited to those genes related to CLL (Supplementary Table 1), we found a 4.88-fold (95% CI: 2.44–9.76, *P* < 0.001, Table 1) increased risk of lymphoid malignancy. In a multivariate model that included both MBL and CHIP, as well as age and sex, MBL and CHIP both remained consistent and significantly associated with incident lymphoid malignancies (Table 1).

**Table 1:** Association between clonal hematopoiesis of indeterminate potential (CHIP) and monoclonal B-cell lymphocytosis (MBL) with incident lymphoid or myeloid malignancy.

	Single precursor models^a^				Multivariable model^a^ with MBL and CHIP	
Precursor	No precursor of interest: Total (Events)	Precursor of interest: Total (Events)	HR (95% CI)	*P*-Value	HR (95% CI)	*P*-Value
**Lymphoid malignancy**						
MBL	8553 (57)	1514 (37)	3.48 (2.27–5.34)	<0.001	3.46 (2.25–5.31)	<0.001
LC-MBL	8553 (57)	1466 (27)	2.62 (1.63–4.20)	<0.001	2.61 (1.63–4.18)	<0.001
CHIP	9200 (78)	867 (16)	1.89 (1.10–3.27)	0.022	1.86 (1.07–3.21)	0.027
CHIP-lymphoid	9900 (87)	167 (7)	4.58 (2.11–9.93)	<0.001	4.73 (2.18–10.26)	<0.001
CHIP-CLL	9865 (85)	202 (9)	4.88 (2.44–9.76)	<0.001	4.88 (2.44–9.76)	<0.001
**Myeloid malignancy**						
MBL	8553 (35)	1514 (11)	1.49 (0.75–2.98)	0.258	1.43 (0.72–2.86)	0.311
CHIP	9200 (28)	867 (18)	6.00 (3.26–11.03)	<0.001	5.95 (3.23–10.94)	<0.001
CHIP-myeloid	9346 (29)	721 (17)	6.62 (3.57–12.28)	<0.001	6.57 (3.54–12.18)	<0.001

CHIP-lymphoid: CHIP variants in genes related to lymphoid. CHIP-myeloid: CHIP variants in genes related to myeloid. CHIP-CLL: CHIP variants in a subset of genes related to CLL.

^a^All models adjusted for age, sex, and cohort.

**Fig. 3:**
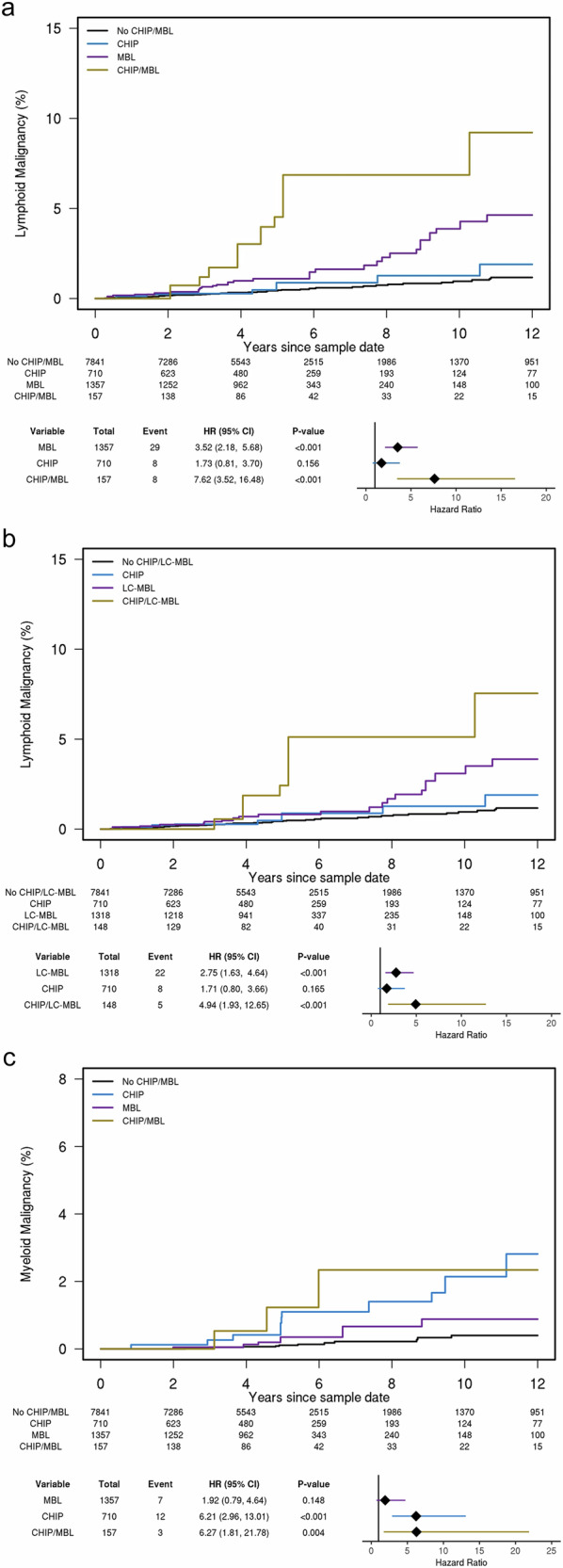
Co-occurrence of monoclonal B-cell lymphocytosis (MBL) and clonal hematopoiesis of indeterminate potential (CHIP) with incident hematological malignancies. CHIP is defined as variants in genes associated with either myeloid (*N* = 56 genes) or lymphoid (*N* = 235 genes). **a** Incident lymphoid malignancy. **b** Incident lymphoid malignancy; however, MBL includes only those with low-count MBL (LC-MBL). **c** Incident myeloid malignancy. The cumulative incidence of malignancy (lymphoid or myeloid) was computed using the Aalen–Johansen methods, where death was considered a competing event.

Although MBL and CHIP are independent predictors, we next investigated the collective effect of MBL and CHIP on the cumulative incidence of lymphoid malignancies. Individuals were categorized into those with no precursors (*N* = 7843, 77.9%), those with CHIP alone (*N* = 710, 7.1%), those with MBL alone (*N* = 1357, 13.5%), or those with both MBL and CHIP (*N* = 157, 1.6%). The 10-year cumulative incidence of lymphoid malignancy was 0.9% (95% CI:0.6–1.3) for individuals with no precursor, 1.2% (95% CI: 0.5–3.1) for individuals with CHIP alone, 3.7% (95% CI: 2.2–6.0) for individuals with MBL alone, and 6.3% (95% CI:2.7–14.5) for those with both MBL and CHIP. Compared to individuals with no precursors, individuals with both CHIP and MBL had a 7.18-fold (95% CI: 3.33–15.47, *P* < 0.001, Fig. 3a) increased risk of lymphoid malignancies; individuals with MBL alone had 3.30-fold (95% CI: 2.06–5.30, *P* < 0.001) increased risk, and individuals with CHIP alone had an elevated but nonsignificant effect (HR = 1.63, 95% CI: 0.77–3.47, *P* = 0.204, Fig. 3a). Of interest, five of the eight incident lymphoid malignancies in those with CHIP only were either CLL or multiple myeloma (MM, Supplementary Table 4), both of which have a necessary precursor condition. The synergistic effect of MBL and CHIP was supported in stratified analyses. Among 1514 individuals with MBL, we found that individuals with both MBL and CHIP had a 2.24-fold (95% CI: 1.01–4.95, *P* = 0.046) increased risk of lymphoid malignancy compared to individuals with MBL but no CHIP. Similarly, among 867 individuals with CHIP, we found that individuals with MBL and CHIP had a 5.02-fold (95% CI: 1.86–13.57, *P* = 0.001) increased risk of lymphoid malignancy compared to individuals with CHIP and no MBL. When analysis was limited to CHIP variants in CLL-related genes (CHIP-CLL), individuals with both CHIP-CLL and MBL (*N* = 45) had a 20.93-fold increased risk of lymphoid malignancies (95% CI: 8.24–53.19, *P* < 0.001, Supplementary Fig. 3) compared to the 3.31-fold risk among individuals with MBL alone (95% CI: 2.10–5.20, *P* < 0.001) and the 3.62-fold risk among individuals with CHIP-CLL alone (95% CI: 1.31–10.06, *P* = 0.014). This resulted in a 10-year cumulative incidence of 18.9% (95% CI: 6.9–51.1) for those with both MBL and CHIP-CLL compared to 0.9% (95% CI: 0.6–1.2) in those with neither of these precursors. Similar results were observed with the co-occurrence of MBL and CHIP in lymphoid-related genes (Supplementary Fig. 4). When evaluating the co-occurrence of LC-MBL and CHIP, those with both precursors had a 4.67-fold (95% CI: 1.83–11.92, *P* = 0.001) and those with LC-MBL alone had 2.58 (95% CI: 1.54–4.33, *P* < 0.001) increased risk of lymphoid malignancy relative to individuals with no precursors (Fig. 3b).

### Association between precursor conditions and myeloid malignancies

A total of 46 incident myeloid malignancies were identified (Supplementary Table 4). In multivariate analyses, when evaluating the effect of MBL, an elevated but non-significant association with incident myeloid malignancies was observed (HR = 1.49, 95% CI: 0.75–2.98, *P* = 0.258, Table 1). In contrast, CHIP was strongly associated with risk of incident myeloid malignancies (HR = 6.00, 95% CI: 3.26–11.03, *P* < 0.001, Table 1). This association remained consistent when the presence of MBL was included in the model (Table 1). When analysis was limited to CHIP variants in genes related to myeloid malignancy, the association with incident myeloid malignancies increased (HR = 6.62, 95% CI: 3.57–12.28, *P* < 0.001, Table 1). When modeling the co-occurrence of CHIP and MBL on incident myeloid malignancies, individuals with both MBL and CHIP (HR = 7.33, 95% CI: 2.45–21.93, *P* < 0.001, Fig. 3c) had statistically similar risk as individuals with CHIP alone (HR = 6.61, 95% CI: 3.31–13.21, *P* < 0.001) relative to individuals with no precursors.

## Discussion

This study, encompassing over 10,000 individuals, represents the largest study to date evaluating the prevalence of both MBL and CHIP within the same cohort. Our findings revealed no evidence of an association between CHIP and MBL, highlighting that these conditions are distinct precursor states. In a previous study of 918 individuals evaluated by targeted sequencing with >1000x coverage, we found that MBL and CHIP based on 42 myeloid-related genes were independent conditions [31]. This finding was confirmed in the current study, which analyzed a cohort ten times larger and included the 918 individuals from the prior study. For the first time, the present study evaluated the association of MBL with CHIP mutations in lymphoid-related genes (termed L-CHIP by Niroula et al. [17]), a condition strongly linked to the development of lymphoid malignancies [17]. Our results revealed that MBL and L-CHIP remained independent of each other.

While previous studies have reported that either MBL or CHIP (particularly, L-CHIP) is associated with increased lymphoid malignancy risk, they did not account for the presence of the other precursor condition. The present study was uniquely positioned to evaluate the relationship between both MBL and CHIP and their risk of incident lymphoid malignancy. In a prior study of 1651 individuals, including 193 with CLL-type MBL, we reported a significant 6.8-fold association between MBL and incident lymphoid malignancy [9]. The current study confirms these findings in a much larger cohort of over 10,000 individuals, including 1514 with CLL-type MBL, with a robust 3.5-fold significant association. Notably, we also demonstrated for the first time that MBL is associated with a 27-fold risk of CLL, specifically, and a 2.3-fold risk of incident lymphoid malignancies other than CLL, indicating that MBL is not only a precursor to CLL but also an important biomarker for other lymphoid malignancies. Additionally, we validated earlier reports that CHIP, and L-CHIP in particular, are significantly associated with incident lymphoid malignancies, after adjusting for MBL in the model [17].

Although our findings underscored the distinct and independent contributions of MBL and CHIP to the development of lymphoid malignancy, we observed a synergistic impact on risk when they co-occurred. Specifically, the risk of incident lymphoid malignancy in individuals with both MBL and CHIP was more than double (7.2-fold) that of individuals with MBL alone (3.3-fold). When limiting analyses to CHIP variants in CLL-related genes in combination with MBL, the effect on developing lymphoma dramatically increased by more than 6-fold (i.e., from 3.3-fold risk with MBL alone to 20.9-fold risk with the co-occurrence of MBL and CLL-related CHIP). This potential synergistic interaction between MBL and CHIP was supported in stratified analyses in individuals with MBL only and indirectly observed in Niroula et al. [17], in that they found more than a 500-fold elevated risk of CLL in individuals with L-CHIP who had lymphocytosis (a presumed surrogate for HC-MBL). In contrast, the risk of lymphoid malignancy associated with CHIP alone attenuated and was no longer statistically significant once those with co-existent MBL were removed. Of the eight individuals with CHIP alone who progressed to lymphoid malignancy, five developed either MM or CLL, both of which have a necessary precursor, suggesting that CHIP may not be an independent driver of lymphoid malignancy. The strong evidence of synergy suggests that these two independent clones, CHIP and MBL, are biologically interacting with each other, and further research is needed to elucidate this interaction.

Another profound finding of the present study was our ability to evaluate individuals with LC-MBL, a condition that affects 8–10 million adults in the United States and is >30 times more common than HC-MBL. We found that co-occurrence of LC-MBL and CHIP was associated with a significant 4.7-fold increased risk of lymphoid malignancy compared to a significant 2.6-fold risk among those with LC-MBL alone. Collectively, these findings suggest that CHIP alone may not be sufficient for progression to lymphoid malignancy and that MBL, or another hematological precursor, may be necessary. Conversely, individuals with MBL have a significantly elevated risk of lymphoid malignancy in the absence of CHIP, yet the presence of CHIP confers an even further enhanced risk of progression in individuals with MBL.

Consistent with previous research, our study independently confirmed the strong association between CHIP mutations in myeloid-related genes and the increased risk of myeloid malignancy [16, 17]. Notably, MBL did not impact the risk of myeloid malignancy, suggesting the observed synergistic effect of MBL and CHIP on lymphoid malignancy did not hold true for the risk of developing myeloid malignancies.

Lymphoid malignancies are relatively rare in the general population, and this study does not support general population screening for MBL or CHIP conditions. However, CHIP precursor clinics are increasingly being established at medical centers across the United States, including our own institutions. In contrast to the growing number of CHIP clinics, MBL screening among asymptomatic individuals is currently limited to research settings, and clinical testing for MBL typically only occurs among individuals with lymphocytosis. Based on our study, individuals who have been referred to a CHIP clinic may benefit from MBL screening to more accurately inform their risk of developing a lymphoid malignancy. Specifically, based on the present findings indicating a 7.2-fold increased risk of lymphoid malignancy in individuals with co-existent CHIP and MBL, we recommend that MBL screening be considered among individuals with CHIP, especially if the CHIP variants occurred in genes also related to CLL (e.g., *TP53, SF3B1*, and *ASXL1*). Herein, we found that individuals with both precursors (i.e., MBL and CHIP-CLL) have a 19% (~1 in 5) chance of developing lymphoid malignancy in 10 years relative to just 1% (~1 in 110) in those individuals without these precursors. Conversely, it is premature to make clinical recommendations regarding CHIP screening in individuals with clinically detected MBL since the marked elevation in risk of lymphoid malignancy is already present among individuals with MBL regardless of CHIP status. Precursor clinics may be the ideal venue for the management of these individuals.

Strengths of this study include the large cohort of individuals who underwent both MBL and CHIP screening, with results of both screening assays unknown to study participants, the median follow-up of five years to identify incident hematological cancers, and our manual review process to confirm incident hematological malignancies. Importantly, the determination of MBL and CHIP status was conducted independently by separate individuals who were blinded to the status of the other precursor. Similarly, the individuals abstracting hematological malignancy were blinded to MBL and CHIP status. One limitation of our study is that while individuals in Cohort 1 had CHIP and MBL performed on the same blood draw, individuals in Cohort 2 had MBL screening conducted ~10 years after CHIP screening. This time gap could have resulted in missing a fraction of individuals who subsequently developed CHIP prior to screening for MBL, leading to misclassification of a small number of individuals as CHIP-negative. This misclassification, however, would bias the results towards the null hypothesis and, therefore, underestimate the association between CHIP and the risk of incident hematological malignancies or the synergistic impact of MBL and CHIP co-occurrence. Another limitation is the lack of biological confirmation of the observed independence between MBL and CHIP or that the CHIP variants originated in progenitor cells. Future studies utilizing single-cell sequencing could help address these gaps. We acknowledge that our WES approach has limited sensitivity for detecting small CHIP clones. While our prior work using targeted sequencing with >1000x coverage found no evidence of an association between MBL and CHIP, the potential impact of undetected small clones on the risk of progression to hematological malignancy needs further study. Finally, the confidence intervals for associations with progression to lymphoid malignancy were wide, indicating low precision in our estimates.

In conclusion, this large screening cohort of over 10,000 individuals demonstrated that MBL and CHIP are distinct and independent precursor conditions. The present study confirmed a strong association between MBL and risk of incident lymphoid malignancy, including increased risk of lymphoid malignancies other than CLL. More profoundly, the presence of CHIP, particularly CLL-related CHIP, significantly amplified the risk of incident lymphoid malignancy among individuals with MBL, whereas CHIP alone may be insufficient to drive progression to lymphoid malignancy. Collectively, these findings underscore the importance of considering both conditions in risk stratification and could enhance early detection and improve clinical management of high-risk individuals with both conditions.

## Supplementary information

Supplemental files

Supplementary figure legend

## Data Availability

Available from the corresponding author upon reasonable request.
