# Supplementary material for: CHIP amplifies the risk of lymphoid malignancies in individuals with monoclonal B-cell lymphocytosis (MBL)
Source: Blood Cancer J. 2025 Nov 6;15(1):195. doi: 10.1038/s41408-025-01385-8 (PMC12592468; doi:10.1038/s41408-025-01385-8)
Supplement: Supplementary file 1 — Supplemental files [file 41408_2025_1385_MOESM1_ESM.pdf]

Supplementary Data

1. Supplementary Methods
2. Supplementary References
3. Supplementary Tables
4. Supplementary Figures

MBL screening

Across the two cohorts, frozen peripheral blood mononuclear cells were screened for MBL using eight-color flow cytometry (CD19, CD23, CD5, CD38, CD45, Kappa, Lambda, and CD20) with the capacity to detect clonal B-cell events with a sensitivity of 0.005% (1/20,000 events). MBL immunophenotype was categorized as follows: (1) CLL-type MBL (CD5+ CD20^{dim} or CD5+ $\text{CD20}^{\text{bright}}$) and (2) non-CLL-type MBL (CD5- $\text{CD20}^{\text{bright}}$). Individuals whose major clone immunophenotype was non-CLL-type MBL were excluded from this study. As validated in our previous studies, individuals with MBL were classified as LC-MBL if <85% of B-cells were clonal B-cells and HC-MBL if ≥85% of B-cells were clonal B-cells.[1,2]

CHIP screening

CHIP screening was performed on DNA from whole blood using whole exome sequencing (WES) a mean coverage depth of 48X. The targeted bases were captured using the Twist Comprehensive Exome design. The captured libraries were sequenced on the Illumina NovaSeq 6000 system on S4 flow cells using paired-end 75 bp reads. CRAM files were read through MUTECT2 to identify somatic mutations. CHIP variants were excluded if the total number of reads was <20, the number of supporting variant allele reads was <3, the variant allele frequency (VAF) was <2%, or alternate reads were reported only on one strand. Variants with a gnomAD frequency ≥0.005 were excluded. CHIP was called and included all protein truncating and missense mutations in known hotspot regions in the 291 genes (**Supplemental Table 1**) related to myeloid (N=56) or lymphoid malignancy (N=235), per Niroula et al.[3] In a subsample of the 1,023

individuals in the MBL cohort, we performed targeted sequencing on whole blood DNA of 42-CHIP related genes.[4] In brief, samples were sequenced using the Illumina HiSeq 4000 with the coverage depth of >1000x. Using this targeted sequencing data, 353 CHIP variants with VAF>2% were identified. Of these, 132 (37%) were identified in the WES data and 221 (63%) were not identified in the WES data. Of the 221 variants, 210 (95%) had VAF<10% in targeted sequencing, demonstrating that most of the missed variants in WES were small CHIP clones.

References

1. Shanafelt TD, Kay NE, Jenkins G, et al. B-cell count and survival: differentiating chronic lymphocytic leukemia from monoclonal B-cell lymphocytosis based on clinical outcome. Blood 2009;113(18):4188-96. DOI: 10.1182/blood-2008-09-176149.
2. Slager SL, Lanasa MC, Marti GE, et al. Natural history of monoclonal B-cell lymphocytosis among relatives in CLL families. Blood 2021;137(15):2046-2056. DOI: 10.1182/blood.2020006322.
3. Niroula A, Sekar A, Murakami MA, et al. Distinction of lymphoid and myeloid clonal hematopoiesis. Nat Med 2021;27(11):1921-1927. DOI: 10.1038/s41591-021-01521-4.
4. Boddicker NJ, Parikh SA, Norman AD, et al. Relationship among three common hematological premalignant conditions. Leukemia 2023. DOI: 10.1038/s41375-023-01914-z.

Supplemental Table 1: Genes investigated for somatic mutations and type of variant

Gene	CHIP Gene Type	Variants considered for CHIP	CLL-Gene
ABL1	lymphoid	Frameshift/stop gain/splice site, Nonsynonymous	
ACTB	lymphoid	Frameshift/stop gain/splice site, Nonsynonymous	
ACTG1	lymphoid	Splice site	
ADD2	lymphoid	Frameshift/stop gain/splice site, Nonsynonymous	
ADGRV1	lymphoid	Frameshift/stop gain/splice site, Nonsynonymous	
AKAP6	lymphoid	Frameshift/stop gain/splice site, Nonsynonymous	
ALK	lymphoid	Frameshift/stop gain/splice site, Nonsynonymous	
ANKRD50	lymphoid	Frameshift/stop gain/splice site, Nonsynonymous	
AOC2	lymphoid	Frameshift/stop gain/splice site, Nonsynonymous	
ARHGEF1	lymphoid	Frameshift/stop gain/splice site, Nonsynonymous	
ARID1A	lymphoid	Frameshift, nonsense, splice site, AAS G2087R, Nonsynonymous	Yes
ARID1B	lymphoid	Frameshift, nonsense, splice site	
ARID5B	lymphoid	Frameshift, nonsense, splice site	
ATM	lymphoid	Frameshift, nonsense, splice site, AAS R337H, R2832H/C, N2875S, I2888T, R3008C/H, Nonsynonymous	Yes
ATR	lymphoid	Frameshift, nonsense, splice site	
AUTS2	lymphoid	Frameshift/stop gain/splice site, Nonsynonymous	
B2M	lymphoid	Frameshift, nonsense, splice site, AAS L7V L12P/Q/R, Nonsynonymous	
BAZ2A	lymphoid	Frameshift/stop gain/splice site, Nonsynonymous	Yes
BAZ2B	lymphoid	Frameshift/stop gain/splice site, Nonsynonymous	
BCL10	lymphoid	Frameshift, nonsense, splice site	
BCL11A	lymphoid	Frameshift/stop gain/splice site, Nonsynonymous	
BCL2	lymphoid	AAS K22N/R, G33R/E/A, P95A/S/L/R, G101V, A131V/D/G/T, N172D/S, Nonsynonymous	
BCL6	lymphoid	AAS R618C, Nonsynonymous	
BCL7A	lymphoid	Frameshift/stop gain/splice site, Nonsynonymous	
BIRC3	lymphoid	Frameshift, nonsense, splice site	Yes
BRCA1	lymphoid	Frameshift/stop gain/splice site, Nonsynonymous	
BRWD3	lymphoid	Frameshift/stop gain/splice site, Nonsynonymous	
BTG1	lymphoid	Frameshift, nonsense, splice site	
BTG2	lymphoid	Frameshift/stop gain/splice site, Nonsynonymous	
BTK	lymphoid	AAS T316A, Nonsynonymous	Yes
CARD11	lymphoid	AAS C49Y/S, E93D/Q, R113Q, F115I/L/V, T117P/N, g123C/D/S, G126D, F130C/I/V, K215E/M/N/T, D230N,	Yes

		S250P, L251P, R337Q, D357E/V, Y361H, D387A, D401N/V, R423W, E626K, Nonsynonymous	
CCAR1	lymphoid	Frameshift/stop gain/splice site, Nonsynonymous	
CCL4	lymphoid	Frameshift/stop gain/splice site, Nonsynonymous	
CCND1	lymphoid	AAS S41T, V42E, Y44C/D/F/H/N/S, K46E/I/N, C47R/S, T286A, P287S, Nonsynonymous	
CCND3	lymphoid	Frameshift, nonsense, splice site at C-terminal end, AAS T283A/I, P284S/R/A/L/T, D286G, I290K/R/T/M, Nonsynonymous	
CD19	lymphoid	Frameshift/stop gain/splice site, Nonsynonymous	
CD274	lymphoid	Frameshift/stop gain/splice site, Nonsynonymous	
CD58	lymphoid	Frameshift, nonsense, splice site	
CD70	lymphoid	Frameshift/stop gain/splice site, Nonsynonymous	
CD79A	lymphoid	Frameshift/stop gain/splice site, Nonsynonymous	
CD79B	lymphoid	AAS Y196H/S/C/N/D/F, Nonsynonymous	
CD83	lymphoid	Frameshift/stop gain/splice site, Nonsynonymous	
CDKN2A	lymphoid	Frameshift, nonsense, splice site, AAS R112H, Nonsynonymous	
CHD2	lymphoid	Frameshift/stop gain/splice site, Nonsynonymous	Yes
CHD8	lymphoid	Frameshift/stop gain/splice site, Nonsynonymous	
CHEK2	lymphoid	Frameshift, nonsense, splice site	Yes
CIITA	lymphoid	Frameshift, nonsense, splice site, T636M, Nonsynonymous	
CLGN	lymphoid	Frameshift/stop gain/splice site, Nonsynonymous	
CNKSR2	lymphoid	Frameshift/stop gain/splice site, Nonsynonymous	
COL6A3	lymphoid	Frameshift/stop gain/splice site, Nonsynonymous	
CSF2RB	lymphoid	Frameshift/stop gain/splice site, Nonsynonymous	
CTSS	lymphoid	Frameshift/stop gain/splice site, Nonsynonymous	
CXCR4	lymphoid	Frameshift, nonsense, splice site at C-terminal end, Nonsynonymous	
DAZAP1	lymphoid	Frameshift/stop gain/splice site, Nonsynonymous	
DDX11	lymphoid	Frameshift/stop gain/splice site, Nonsynonymous	
DDX3X	lymphoid	Frameshift, nonsense, splice site, AAS Y525H, R528H, R534H/C, Nonsynonymous	Yes
DGKB	lymphoid	Frameshift/stop gain/splice site, Nonsynonymous	
DIRAS3	lymphoid	Frameshift/stop gain/splice site, Nonsynonymous	
DLC1	lymphoid	Frameshift/stop gain/splice site, Nonsynonymous	
DMD	lymphoid	Frameshift/stop gain/splice site, Nonsynonymous	
DTX1	lymphoid	Frameshift, nonsense, splice site	
DUSP2	lymphoid	Frameshift/stop gain/splice site, Nonsynonymous	
DUSP22	lymphoid	Frameshift/stop gain/splice site, Nonsynonymous	

EBF1	lymphoid	Frameshift/stop gain/splice site, Nonsynonymous	
EEF1A1	lymphoid	Frameshift/stop gain/splice site, Nonsynonymous	
ENPP3	lymphoid	Frameshift/stop gain/splice site, Nonsynonymous	
ENTPD4	lymphoid	Frameshift/stop gain/splice site, Nonsynonymous	
ERBB4	lymphoid	AAS K1223T, Nonsynonymous	
ETS1	lymphoid	Frameshift/stop gain/splice site, Nonsynonymous	
FANCE	lymphoid	Frameshift/stop gain/splice site, Nonsynonymous	
FAS	lymphoid	Frameshift, nonsense, splice site	
FAT1	lymphoid	Frameshift, nonsense, splice site, AAS R1627Q, A4419V, Nonsynonymous	
FAT2	lymphoid	Frameshift/stop gain/splice site, Nonsynonymous	
FAT4	lymphoid	Frameshift/stop gain/splice site, Nonsynonymous	
FBXW7	lymphoid	Frameshift, nonsense, splice site, AAS R465H/C/L, Y545C, Nonsynonymous	Yes
FOXC1	lymphoid	Frameshift/stop gain/splice site, Nonsynonymous	
FOXO1	lymphoid	Frameshift, nonsense, splice site, AAS S22P/W, T24I/A, S205N, Nonsynonymous	
FYN	lymphoid	Frameshift/stop gain/splice site, Nonsynonymous	
GNA13	lymphoid	Frameshift/stop gain/splice site, Nonsynonymous	
GNAI2	lymphoid	Frameshift/stop gain/splice site, Nonsynonymous	
GNE	lymphoid	Frameshift/stop gain/splice site, Nonsynonymous	
GPS2	lymphoid	Frameshift, nonsense, splice site	
GRB2	lymphoid	Frameshift/stop gain/splice site, Nonsynonymous	
GRHPR	lymphoid	Frameshift/stop gain/splice site, Nonsynonymous	
GRM7	lymphoid	Frameshift/stop gain/splice site, Nonsynonymous	
HAVCR2	lymphoid	Frameshift/stop gain/splice site, Nonsynonymous	
HEATR3	lymphoid	Frameshift/stop gain/splice site, Nonsynonymous	
HIST1H1B	lymphoid	Frameshift, nonsense, splice site	Yes
HIST1H1C	lymphoid	AAS A180P, Nonsynonymous	
HIST1H1D	lymphoid	Frameshift/stop gain/splice site, Nonsynonymous	
HIST1H1E	lymphoid	Frameshift/stop gain/splice site, Nonsynonymous	Yes
HIST1H2AC	lymphoid	Frameshift/stop gain/splice site, Nonsynonymous	
HIST1H2AM	lymphoid	Frameshift/stop gain/splice site, Nonsynonymous	
HIST1H2BC	lymphoid	AAS E77G/K, A78P, L103F/I, Nonsynonymous	
HIST1H2BK	lymphoid	Frameshift/stop gain/splice site, Nonsynonymous	
HIST1H3B	lymphoid	Frameshift/stop gain/splice site, Nonsynonymous	
HIST2H2BE	lymphoid	Frameshift/stop gain/splice site, Nonsynonymous	
HNRNPD	lymphoid	Frameshift/stop gain/splice site, Nonsynonymous	
HVCN1	lymphoid	Frameshift/stop gain/splice site, Nonsynonymous	

ID3	lymphoid	Frameshift, nonsense, splice site, AAS P56S/L/R, L64F/H/R/V, Nonsynonymous	
IGLL5	lymphoid	Frameshift/stop gain/splice site, Nonsynonymous	
IKZF3	lymphoid	Frameshift/stop gain/splice site, Nonsynonymous	Yes
IL10RA	lymphoid	Frameshift/stop gain/splice site, Nonsynonymous	
IL6	lymphoid	Frameshift/stop gain/splice site, Nonsynonymous	
IRF2BP2	lymphoid	Frameshift/stop gain/splice site, Nonsynonymous	
IRF4	lymphoid	AAS C99R, Nonsynonymous	Yes
IRF8	lymphoid	Frameshift, nonsense, splice site at C-terminal end, AAS T80A, Nonsynonymous	
ITK	lymphoid	Frameshift/stop gain/splice site, Nonsynonymous	
ITPKB	lymphoid	Frameshift/stop gain/splice site, Nonsynonymous	Yes
ITPR3	lymphoid	Frameshift/stop gain/splice site, Nonsynonymous	
JAK1	lymphoid	AAS Y652H, L910P, Y1035C, Nonsynonymous	
JAK3	lymphoid	AAS 573V, R657Q, Nonsynonymous	
KIAA1671	lymphoid	Frameshift/stop gain/splice site, Nonsynonymous	
KIR2DL3	lymphoid	Frameshift/stop gain/splice site, Nonsynonymous	
KLF2	lymphoid	Frameshift, nonsense, splice site	
KLHL14	lymphoid	Frameshift/stop gain/splice site, Nonsynonymous	
KLHL21	lymphoid	Frameshift/stop gain/splice site, Nonsynonymous	
KLHL6	lymphoid	Frameshift/stop gain/splice site, Nonsynonymous	Yes
KLHL9	lymphoid	Frameshift/stop gain/splice site, Nonsynonymous	
KMT2C	lymphoid	Frameshift, nonsense, splice site	
KMT2D	lymphoid	Frameshift, nonsense, splice site, AAS R5179C, R5432W/Q, Nonsynonymous	Yes
LRP1B	lymphoid	Frameshift/stop gain/splice site, Nonsynonymous	
LTB	lymphoid	Frameshift/stop gain/splice site, Nonsynonymous	
LYN	lymphoid	Frameshift/stop gain/splice site, Nonsynonymous	
MAGT1	lymphoid	Frameshift/stop gain/splice site, Nonsynonymous	
MAP2K1	lymphoid	Frameshift/stop gain/splice site, Nonsynonymous	Yes
MATN2	lymphoid	Frameshift/stop gain/splice site, Nonsynonymous	
MCL1	lymphoid	Frameshift/stop gain/splice site, Nonsynonymous	
MED11	lymphoid	Frameshift/stop gain/splice site, Nonsynonymous	
MEF2B	lymphoid	AAS K4E, Y69N, E77K, N81K/Y, D83V, Nonsynonymous	
MGA	lymphoid	Frameshift, nonsense, splice site	Yes
MGARP	lymphoid	Frameshift/stop gain/splice site, Nonsynonymous	
MPEG1	lymphoid	Frameshift/stop gain/splice site, Nonsynonymous	
MS4A1	lymphoid	Frameshift/stop gain/splice site, Nonsynonymous	

MTOR	lymphoid	AAS W1456G, A1459V, C1483Y, A1971T, T1977K, T1977R, V2006I, S2215F, R2217W, S2231L, I2500F, Nonsynonymous	
MYC	lymphoid	AAS V7L/A/M, T73I/N/A, P74S/A, S161L, Nonsynonymous	
MYCBP2	lymphoid	Frameshift/stop gain/splice site, Nonsynonymous	
MYD88	lymphoid	AAS M232T, S243N, L265P	Yes
NBPF14	lymphoid	Frameshift/stop gain/splice site, Nonsynonymous	
NBPF15	lymphoid	Frameshift/stop gain/splice site, Nonsynonymous	
NCOR1	lymphoid	Frameshift, nonsense, splice site	
NCOR2	lymphoid	Frameshift/stop gain/splice site, Nonsynonymous	
NEB	lymphoid	Frameshift/stop gain/splice site, Nonsynonymous	
NFKBIA	lymphoid	Frameshift, nonsense, splice site	
NFKBIE	lymphoid	Frameshift/stop gain/splice site, Nonsynonymous	Yes
NFKBIZ	lymphoid	Frameshift/stop gain/splice site, Nonsynonymous	
NLRP8	lymphoid	Frameshift/stop gain/splice site, Nonsynonymous	
NOL9	lymphoid	Frameshift/stop gain/splice site, Nonsynonymous	
NOTCH1	lymphoid	Frameshift, nonsense, splice site at C-terminal end	Yes
NOTCH2	lymphoid	Frameshift, nonsense, splice site at C-terminal end	
NSD2	lymphoid	AAS E1099K, T1150A	
NTRK1	lymphoid	Frameshift/stop gain/splice site, Nonsynonymous	
NUP214	lymphoid	Frameshift/stop gain/splice site, Nonsynonymous	
OR2M3	lymphoid	Frameshift/stop gain/splice site, Nonsynonymous	
OSBPL10	lymphoid	Frameshift/stop gain/splice site, Nonsynonymous	
P2RY8	lymphoid	Frameshift/stop gain/splice site, Nonsynonymous	
PABPC3	lymphoid	Frameshift/stop gain/splice site, Nonsynonymous	
PAX5	lymphoid	Frameshift, nonsense, splice site, AAS V26G, Nonsynonymous	Yes
PCBP1	lymphoid	Frameshift/stop gain/splice site, Nonsynonymous	
PCLO	lymphoid	Frameshift/stop gain/splice site, Nonsynonymous	
PDCD1	lymphoid	Frameshift/stop gain/splice site, Nonsynonymous	
PDE4DIP	lymphoid	Frameshift/stop gain/splice site, Nonsynonymous	
PDGFRB	lymphoid	Frameshift/stop gain/splice site, Nonsynonymous	
PEX14	lymphoid	Frameshift/stop gain/splice site, Nonsynonymous	
PIK3CA	lymphoid	AAS R108H, Nonsynonymous	
PIK3CD	lymphoid	AAS G124D, C416R, E525K, E1021K, Nonsynonymous	
PIM1	lymphoid	AAS L2F, N7K, K24N, G28D/A/S/V, Q37H, H68Y, E79D/K, P81S/A/I/T, S97N/T, L193F/I, Nonsynonymous	Yes
PIM2	lymphoid	Frameshift/stop gain/splice site, Nonsynonymous	
PLCG2	lymphoid	Frameshift/stop gain/splice site, Nonsynonymous	Yes
PLXNB3	lymphoid	Frameshift/stop gain/splice site, Nonsynonymous	

POT1	lymphoid	Frameshift, nonsense, splice site	Yes
POU2AF1	lymphoid	Frameshift/stop gain/splice site, Nonsynonymous	
POU2F2	lymphoid	Frameshift/stop gain/splice site, Nonsynonymous	
PPP4R3A	lymphoid	Frameshift/stop gain/splice site, Nonsynonymous	
PRDM1	lymphoid	Frameshift, nonsense, splice site	
PRKCB	lymphoid	Frameshift/stop gain/splice site, Nonsynonymous	
PRUNE2	lymphoid	Frameshift/stop gain/splice site, Nonsynonymous	
PTCH1	lymphoid	Frameshift/stop gain/splice site, Nonsynonymous	
PTEN	lymphoid	Frameshift, nonsense, splice site, AAS S10N, L23S, D24E, Y27N, L42F, V45I, H123Y, G129R, I135T, Y155S, R335Q, Nonsynonymous	
PTPN6	lymphoid	Frameshift/stop gain/splice site, Nonsynonymous	
PTPRD	lymphoid	Frameshift, nonsense, splice site, AAS P1311S, V1565I, R1674H, Nonsynonymous	
PTPRK	lymphoid	Frameshift/stop gain/splice site, Nonsynonymous	
RB1	lymphoid	Frameshift, nonsense, splice site, AAS R661W, C706R, S758L, Nonsynonymous	
RBMXL1	lymphoid	Frameshift/stop gain/splice site, Nonsynonymous	
RCOR1	lymphoid	Frameshift/stop gain/splice site, Nonsynonymous	
REL	lymphoid	Frameshift/stop gain/splice site, Nonsynonymous	
RET	lymphoid	Frameshift/stop gain/splice site, Nonsynonymous	
RHOA	lymphoid	AAS R5Q/W, G17A, Y34N, N41S, Y42C/F/S/H, Y66N, L69P/R, A161T, Nonsynonymous	
ROS1	lymphoid	Frameshift/stop gain/splice site, Nonsynonymous	
RP1L1	lymphoid	Frameshift/stop gain/splice site, Nonsynonymous	
RPS15	lymphoid	Frameshift/stop gain/splice site, Nonsynonymous	Yes
SCG3	lymphoid	Frameshift/stop gain/splice site, Nonsynonymous	
SEL1L3	lymphoid	Frameshift/stop gain/splice site, Nonsynonymous	
SETD1B	lymphoid	Frameshift/stop gain/splice site, Nonsynonymous	
SETD5	lymphoid	Frameshift/stop gain/splice site, Nonsynonymous	
SETDB1	lymphoid	Frameshift/stop gain/splice site, Nonsynonymous	
SGK1	lymphoid	Frameshift/stop gain/splice site, Nonsynonymous	
SIN3A	lymphoid	Frameshift/stop gain/splice site, Nonsynonymous	
SLC9A6	lymphoid	Frameshift/stop gain/splice site, Nonsynonymous	
SMARCA4	lymphoid	Frameshift, nonsense, splice site, AAS T910M/A, R973Q/L, R1135Q, R1189L, R1192C/H, R1243L/W, Nonsynonymous	
SOCS1	lymphoid	Frameshift, nonsense, splice site, AAS A3T/S/P/V, Nonsynonymous	
SOCS6	lymphoid	Frameshift/stop gain/splice site, Nonsynonymous	
SPEN	lymphoid	Frameshift, nonsense, splice site	

SPIB	lymphoid	Frameshift/stop gain/splice site, Nonsynonymous	
STAT3	lymphoid	AAS in SH2 domain (S614N/R, E616G/K, Y640F, K658R, D661V/Y), Nonsynonymous	
STAT6	lymphoid	Frameshift/stop gain/splice site, Nonsynonymous	
SYNE1	lymphoid	Frameshift/stop gain/splice site, Nonsynonymous	Yes
TAF1	lymphoid	Frameshift/stop gain/splice site, Nonsynonymous	
TBL1XR1	lymphoid	AAS D370N, W376C, Y395C/H, Nonsynonymous	
TCL1A	lymphoid	Frameshift/stop gain/splice site, Nonsynonymous	
TCTN2	lymphoid	Frameshift/stop gain/splice site, Nonsynonymous	
TLR2	lymphoid	Frameshift/stop gain/splice site, Nonsynonymous	
TMEM30A	lymphoid	Frameshift/stop gain/splice site, Nonsynonymous	
TMSB4X	lymphoid	Frameshift/stop gain/splice site, Nonsynonymous	
TNFAIP3	lymphoid	Frameshift, nonsense, splice site	
TNFRSF14	lymphoid	Frameshift, nonsense, splice site	
TNIP1	lymphoid	Frameshift/stop gain/splice site, Nonsynonymous	
TOX	lymphoid	Frameshift/stop gain/splice site, Nonsynonymous	
TP63	lymphoid	Frameshift/stop gain/splice site, Nonsynonymous	
TRAF3	lymphoid	Frameshift/stop gain/splice site, Nonsynonymous	Yes
TSC2	lymphoid	Frameshift, nonsense, splice site	
TUT4	lymphoid	Frameshift/stop gain/splice site, Nonsynonymous	
UBE2A	lymphoid	Frameshift/stop gain/splice site, Nonsynonymous	
UBR5	lymphoid	Frameshift/stop gain/splice site, Nonsynonymous	
USP6	lymphoid	Frameshift/stop gain/splice site, Nonsynonymous	
VAV1	lymphoid	Frameshift/stop gain/splice site, Nonsynonymous	
WDFY3	lymphoid	Frameshift/stop gain/splice site, Nonsynonymous	
XPO1	lymphoid	AAS E571K/V/Q/G, Nonsynonymous	Yes
YY1	lymphoid	Frameshift/stop gain/splice site, Nonsynonymous	
ZBTB11	lymphoid	Frameshift/stop gain/splice site, Nonsynonymous	
ZC3H12A	lymphoid	Frameshift/stop gain/splice site, Nonsynonymous	
ZEB2	lymphoid	Frameshift/stop gain/splice site, Nonsynonymous	
ZFP36L1	lymphoid	Frameshift/stop gain/splice site, Nonsynonymous	
ZNF217	lymphoid	Frameshift/stop gain/splice site, Nonsynonymous	
ZNF292	lymphoid	Frameshift/stop gain/splice site, Nonsynonymous	Yes
ASXL1	Myeloid	Frameshift/stop gain/splice site in exon 11-12	Yes
ASXL2	Myeloid	Frameshift/stop gain/splice site in exon 11-12	
BCOR	Myeloid	Frameshift/stop gain/splice site	Yes
BCORL1	Myeloid	Frameshift/stop gain/splice site	
BRAF	Myeloid	Nonsynonymous AA469, AA590-610	Yes
BRCC3	Myeloid	Frameshift/stop gain/splice site	Yes
CALR	Myeloid	Frameshift (C-terminal) AA352-418	

CBL	Myeloid	Frameshift/stop gain/splice site/nonsynonymous AA360-430	
CEBPA	Myeloid	Frameshift/stop gain	
CREBBP	Myeloid	Frameshift/stop gain/splice site, nonysnonymous AA1446	Yes
CSF3R	Myeloid	Frameshift/stop gain AA618-840, Nonsynonymous AA615, AA618	
CTCF	Myeloid	Frameshift/stop gain/splice site	
CUX1	Myeloid	Frameshift/stop gain/splice site	
DNMT3A	Myeloid	Frameshift/stop gain/splice site, Nonsynonymous AA290-390, AA547-911, AA414, AA494, AA497, AA508, AA527, AA529, AA531, AA532, AA537, AA543	
EED	Myeloid	Frameshift/stop gain/splice site, Nonsynonymous AA196/240, AA363	
EP300	Myeloid	Frameshift/stop gain/splice site	
ETNK1	Myeloid	Nonsynonymous AA243-245	
ETV6	Myeloid	Frameshift/stop gain/splice site	
EZH2	Myeloid	Frameshift/stop gain/splice site, Nonsynonymous AA500-738	
FLT3	Myeloid	Nonsynonymous AA800-850, AA569-700	
GATA2	Myeloid	Frameshift/stop gain/splice site, Nonsynonymous AA250-398	
GNAS	Myeloid	Nonsynonymous AA201, AA227, AA844	
GNB1	Myeloid	Nonsynonymous AA57, AA76, AA77, AA78, AA80, AA81, AA88, AA89	Yes
IDH1	Myeloid	Nonsynonymous AA126-138	
IDH2	Myeloid	Nonsynonymous AA134-146, 164-180	
IKZF1	Myeloid	Frameshift/stop gain/splice site	
JAK2	Myeloid	Nonsynonymous AA505-547, AA617, AA682, AA683	
KDM6A	Myeloid	Frameshift/stop gain/splice site	
KIT	Myeloid	Nonsynonymous AA400-430, AA540-600, AA810-830	
KRAS	Myeloid	Nonsynonymous AA5, AA12, AA13, AA18, AA33, AA59, AA60, AA61, AA64, AA117, AA146	Yes
MPL	Myeloid	Nonsynonymous AA500-520	
NF1	Myeloid	Frameshift/stop gain/splice site	
NPM1	Myeloid	Frameshift AA260-300	
NRAS	Myeloid	Nonsynonymous AA12, AA13, AA14, AA18, AA24, AA59, AA60, AA61, AA63, AA64, AA66, AA69, AA74, AA146	Yes
PDS5B	Myeloid	Frameshift/stop gain/splice site	
PHF6	Myeloid	Frameshift/stop gain/splice site AA42-132, AA235-330	

PHIP	Myeloid	Frameshift/stop gain/splice site	
PPM1D	Myeloid	Frameshift/stop gain/splice site AA421-605	
PRPF40B	Myeloid	Frameshift/nonsense/splicesite, Nonsynonymous AA15, AA58, AA405, AA562	
PRPF8	Myeloid	Nonsynonymous AA1591, AA1594, AA1598, AA1603	
PTPN11	Myeloid	Nonsynonymous AA40, AA42, AA45, AA58, AA60, AA61, AA62, AA69, AA71, AA72, AA73, AA76, AA79, AA163, AA186, AA265, AA282, AA296, AA308, AA419, AA424, AA428, AA468, AA491, AA502, AA503, AA504, AA510	Yes
RAD21	Myeloid	Frameshift/stop gain/splice site	
RUNX1	Myeloid	Frameshift/stop gain/splice site, Nonsynonymous AA90-250, AA396	
SETBP1	Myeloid	Nonsynonymous AA855-880	
SETD2	Myeloid	Frameshift/stop gain/splice site	Yes
SF3B1	Myeloid	Nonsynonymous AA550-800	Yes
SMC1A	Myeloid	Frameshift/stop gain/splice site	
SMC3	Myeloid	Frameshift/stop gain/splice site	
SRSF2	Myeloid	Nonsynonymous AA57, AA95, AA85-100	
STAG2	Myeloid	Frameshift/stop gain/splice site	
SUZ12	Myeloid	Frameshift/stop gain/splice site	
TET2	Myeloid	Frameshift/stop gain/splice site, Nonsynonymous AA1104-1481, AA1843-2002	
TP53	Myeloid	Frameshift/stop gain/splice site, Nonsynonymous	Yes
U2AF1	Myeloid	Nonsynonymous AA22, AA28, AA32, AA34, AA154, AA156, AA157	
WT1	Myeloid	Frameshift/stop gain/splice site	
ZRSR2	Myeloid	Frameshift/stop gain/splice site	

Supplemental Table 2. Participant characteristics (N=10,067)

	N (%)
**Cohort**	
Cohort 1	3904 (38.8)
Cohort 2	6163 (61.2)
**Biobank Enrollment Age**	
Median, years (range)	61.7 (28.7, 96.5)
**Age at MBL Sample**	
Median, years (range)	66.2 (40.1-101.3)
**Sex**	
Female	6233 (61.9)
Male	3834 (38.1)
**Genetic Ancestry**	
African	37 (0.4)
Native American	164 (1.6)
East Asian	64 (0.6)
European	9740 (96.8)
South Asian	27 (0.3)
Mixed	31 (0.3%)
Missing	4
**MBL**	
Negative	8553 (85.0)
Positive	1514 (15.0)
**CHIP**	
No	9200 (91.4)
Yes	867 (8.6)

Supplemental Table 3: CHIP variants identified in WES (hg38)

ID	CHIP Gene Type	CHROM	POS	DP	VAF	Gene	Variant Classification	Variant Type	Protein Change
161	lymphoid	chr9	130885259	62	0.113	ABL1	NSY	Substitution	p.Ser990Leu
103	lymphoid	chr2	70706323	47	0.128	ADD2	NSY	Substitution	p.Pro29His
127	lymphoid	chr5	90642886	60	0.133	ADGRV1	SG	Substitution	p.Arg800X
128	lymphoid	chr5	90750609	67	0.149	ADGRV1	NSY	Substitution	p.Val3678Ala
95	lymphoid	chr17	42845521	38	0.158	AOC2	NSY	Substitution	p.Arg299Trp
13	lymphoid	chr1	26697208	54	0.056	ARID1A	SG	Substitution	p.Gln269X
61	lymphoid	chr1	26729688	40	0.2	ARID1A	NSY	Substitution	p.Pro392His
14	lymphoid	chr1	26731386	53	0.057	ARID1A	SG	Substitution	p.Gln529X
15	lymphoid	chr1	26731527	40	0.075	ARID1A	SG	Substitution	p.Gln576X
16	lymphoid	chr1	26771203	81	0.037	ARID1A	SG	Substitution	p.Gln1095X
17	lymphoid	chr1	26774613	48	0.062	ARID1A	FS	Insertion	
2	lymphoid	chr1	26775118	42	0.071	ARID1A	SG	Substitution	p.Gln1631X
18	lymphoid	chr1	26775575	44	0.068	ARID1A	ESS	Substitution	
19	lymphoid	chr1	26779439	58	0.052	ARID1A	FS	Deletion	
53	lymphoid	chr6	156778444	60	0.067	ARID1B	FS	Deletion	
54	lymphoid	chr6	157084819	55	0.073	ARID1B	FS	Deletion	
198	myeloid	chr20	32431435	64	0.062	ASXL1	SG	Substitution	p.Ser278X
216	myeloid	chr20	32431652	45	0.067	ASXL1	SG	Substitution	p.Gln318X
175	myeloid	chr20	32433408	64	0.25	ASXL1	SG	Substitution	p.Arg404X
206	myeloid	chr20	32433408	53	0.34	ASXL1	SG	Substitution	p.Arg404X
168	myeloid	chr20	32433447	58	0.086	ASXL1	SG	Substitution	p.Arg417X
174	myeloid	chr20	32433447	76	0.053	ASXL1	SG	Substitution	p.Arg417X
178	myeloid	chr20	32433447	75	0.253	ASXL1	SG	Substitution	p.Arg417X
125	myeloid	chr20	32433480	64	0.094	ASXL1	SG	Substitution	p.Gln428X
234	myeloid	chr20	32433732	62	0.242	ASXL1	SG	Substitution	p.Gln512X
170	myeloid	chr20	32433747	59	0.068	ASXL1	SG	Substitution	p.Gln517X
229	myeloid	chr20	32433747	52	0.058	ASXL1	SG	Substitution	p.Gln517X

210	myeloid	chr20	32433750	57	0.088	ASXL1	SG	Substitution	p.Glu518X
184	myeloid	chr20	32433819	84	0.19	ASXL1	FS	Insertion	
235	myeloid	chr20	32434483	38	0.316	ASXL1	FS	Insertion	
185	myeloid	chr20	32434485	40	0.075	ASXL1	SG	Substitution	p.Tyr591X
179	myeloid	chr20	32434494	31	0.129	ASXL1	SG	Substitution	p.Cys594X
177	myeloid	chr20	32434599	51	0.216	ASXL1	FS	Deletion	
186	myeloid	chr20	32434599	57	0.158	ASXL1	FS	Deletion	
205	myeloid	chr20	32434599	74	0.054	ASXL1	FS	Deletion	
224	myeloid	chr20	32434599	68	0.147	ASXL1	FS	Deletion	
230	myeloid	chr20	32434599	71	0.085	ASXL1	FS	Deletion	
233	myeloid	chr20	32434599	67	0.179	ASXL1	FS	Deletion	
172	myeloid	chr20	32434638	30	0.367	ASXL1	FS	Insertion	
176	myeloid	chr20	32434638	42	0.31	ASXL1	FS	Insertion	
180	myeloid	chr20	32434638	55	0.091	ASXL1	FS	Insertion	
181	myeloid	chr20	32434638	37	0.162	ASXL1	FS	Insertion	
182	myeloid	chr20	32434638	78	0.141	ASXL1	FS	Insertion	
187	myeloid	chr20	32434638	46	0.098	ASXL1	FS	Insertion	
188	myeloid	chr20	32434638	37	0.081	ASXL1	FS	Insertion	
189	myeloid	chr20	32434638	69	0.072	ASXL1	FS	Insertion	
190	myeloid	chr20	32434638	37	0.243	ASXL1	FS	Insertion	
193	myeloid	chr20	32434638	47	0.213	ASXL1	FS	Insertion	
194	myeloid	chr20	32434638	38	0.105	ASXL1	FS	Insertion	
196	myeloid	chr20	32434638	38	0.079	ASXL1	FS	Insertion	
197	myeloid	chr20	32434638	31	0.097	ASXL1	FS	Insertion	
200	myeloid	chr20	32434638	52	0.135	ASXL1	FS	Insertion	
202	myeloid	chr20	32434638	64	0.234	ASXL1	FS	Insertion	
204	myeloid	chr20	32434638	64	0.049	ASXL1	FS	Insertion	
207	myeloid	chr20	32434638	54	0.259	ASXL1	FS	Insertion	
208	myeloid	chr20	32434638	48	0.062	ASXL1	FS	Insertion	
97	myeloid	chr20	32434638	45	0.067	ASXL1	FS	Insertion	
209	myeloid	chr20	32434638	54	0.204	ASXL1	FS	Insertion	

211	myeloid	chr20	32434638	42	0.119	ASXL1	FS	Insertion	
212	myeloid	chr20	32434638	51	0.059	ASXL1	FS	Insertion	
214	myeloid	chr20	32434638	49	0.102	ASXL1	FS	Insertion	
215	myeloid	chr20	32434638	84	0.048	ASXL1	FS	Insertion	
217	myeloid	chr20	32434638	39	0.231	ASXL1	FS	Insertion	
219	myeloid	chr20	32434638	41	0.073	ASXL1	FS	Insertion	
220	myeloid	chr20	32434638	64	0.062	ASXL1	FS	Insertion	
223	myeloid	chr20	32434638	31	0.097	ASXL1	FS	Insertion	
146	myeloid	chr20	32434638	79	0.215	ASXL1	FS	Insertion	
226	myeloid	chr20	32434638	34	0.088	ASXL1	FS	Insertion	
227	myeloid	chr20	32434638	56	0.161	ASXL1	FS	Insertion	
228	myeloid	chr20	32434638	50	0.08	ASXL1	FS	Insertion	
231	myeloid	chr20	32434638	46	0.174	ASXL1	FS	Insertion	
236	myeloid	chr20	32434638	54	0.093	ASXL1	FS	Insertion	
237	myeloid	chr20	32434638	38	0.263	ASXL1	FS	Insertion	
183	myeloid	chr20	32434642	51	0.255	ASXL1	FS	Deletion	
203	myeloid	chr20	32434789	42	0.381	ASXL1	SG	Substitution	p.Arg693X
225	myeloid	chr20	32434789	43	0.233	ASXL1	SG	Substitution	p.Arg693X
171	myeloid	chr20	32434809	77	0.221	ASXL1	FS	Deletion	
199	myeloid	chr20	32434864	60	0.3	ASXL1	SG	Substitution	p.Arg718X
201	myeloid	chr20	32434901	51	0.157	ASXL1	FS	Deletion	
221	myeloid	chr20	32434927	39	0.128	ASXL1	FS	Deletion	
173	myeloid	chr20	32435006	49	0.163	ASXL1	FS	Deletion	
232	myeloid	chr20	32435036	32	0.344	ASXL1	SG	Substitution	p.Leu775X
213	myeloid	chr20	32435158	47	0.085	ASXL1	FS	Deletion	
195	myeloid	chr20	32435309	42	0.143	ASXL1	SG	Substitution	p.Leu866X
191	myeloid	chr20	32435328	46	0.065	ASXL1	SG	Substitution	p.Cys872X
218	myeloid	chr20	32435356	44	0.091	ASXL1	SG	Substitution	p.Gln882X
102	myeloid	chr20	32435440	49	0.102	ASXL1	SG	Substitution	p.Gln910X
124	myeloid	chr20	32435641	40	0.075	ASXL1	SG	Substitution	p.Gln977X
169	myeloid	chr20	32435803	75	0.347	ASXL1	SG	Substitution	p.Lys1031X

192	myeloid	chr20	32436440	90	0.033	ASXL1	FS	Deletion	
222	myeloid	chr20	32436592	65	0.046	ASXL1	SG	Substitution	p.Gln1294X
240	myeloid	chr2	25742700	71	0.042	ASXL2	SG	Substitution	p.Gly1213X
241	myeloid	chr2	25744310	37	0.135	ASXL2	FS	Insertion	
242	myeloid	chr2	25753607	51	0.059	ASXL2	SG	Substitution	p.Arg357X
239	myeloid	chr2	25768832	52	0.058	ASXL2	SG	Substitution	p.Gln181X
238	myeloid	chr2	25768850	33	0.091	ASXL2	SG	Substitution	p.Gln175X
25	lymphoid	chr11	108247072	55	0.055	ATM	NSY	Substitution	p.Arg337His
72	lymphoid	chr11	108249096	31	0.194	ATM	NSY	Substitution	p.Val410Ala
73	lymphoid	chr11	108330281	78	0.179	ATM	NSY	Substitution	p.Arg2459Ser
26	lymphoid	chr11	108335929	41	0.171	ATM	FS	Deletion	
27	lymphoid	chr11	108345819	75	0.253	ATM	NSY	Substitution	p.Arg2832His
28	lymphoid	chr11	108345819	119	0.042	ATM	NSY	Substitution	p.Arg2832His
29	lymphoid	chr11	108345859	72	0.167	ATM	SG	Substitution	p.Trp2845X
30	lymphoid	chr11	108365359	49	0.408	ATM	NSY	Substitution	p.Arg3008Cys
31	lymphoid	chr11	108365360	60	0.217	ATM	NSY	Substitution	p.Arg3008His
46	lymphoid	chr3	142449572	20	0.25	ATR	SG	Substitution	p.Arg2598X
40	lymphoid	chr18	63318601	43	0.07	BCL2	NSY	Substitution	p.Lys22Asn
47	lymphoid	chr3	187725066	45	0.067	BCL6	NSY	Substitution	p.Arg618Cys
244	myeloid	chrX	40055470	33	0.091	BCOR	SG	Substitution	p.Arg1547X
246	myeloid	chrX	40062767	69	0.043	BCOR	SG	Substitution	p.Tyr1384X
245	myeloid	chrX	40072425	48	0.062	BCOR	FS	Insertion	
248	myeloid	chrX	40072807	38	0.079	BCOR	SG	Substitution	p.Gln847X
247	myeloid	chrX	40072931	62	0.048	BCOR	SG	Substitution	p.Tyr805X
243	myeloid	chrX	40074884	44	0.159	BCOR	FS	Deletion	
249	myeloid	chrX	130014384	28	0.107	BCORL1	SG	Substitution	p.Gln538X
88	myeloid	chrX	130015767	45	0.067	BCORL1	FS	Deletion	
252	myeloid	chrX	130025190	84	0.036	BCORL1	SG	Substitution	p.Arg1297X
254	myeloid	chrX	130025190	114	0.035	BCORL1	SG	Substitution	p.Arg1297X
251	myeloid	chrX	130025341	27	0.148	BCORL1	FS	Deletion	
253	myeloid	chrX	130028814	67	0.045	BCORL1	SG	Substitution	p.Arg1420X

250	myeloid	chrX	130056035	52	0.058	BCORL1	FS	Deletion	
255	myeloid	chrX	130056035	62	0.048	BCORL1	FS	Deletion	
24	lymphoid	chr11	102336920	99	0.03	BIRC3	SG	Substitution	p.Glu545X
256	myeloid	chrX	155116125	62	0.048	BRCC3	SG	Substitution	p.Ser206X
258	myeloid	chrX	155116163	32	0.125	BRCC3	SG	Substitution	p.Gln219X
257	myeloid	chrX	155120094	61	0.049	BRCC3	SG	Substitution	p.Gln274X
259	myeloid	chr19	12943750	59	0.22	CALR	FS	Deletion	
260	myeloid	chr19	12943762	110	0.1	CALR	FS	Deletion	
143	lymphoid	chr7	2917319	60	0.15	CARD11	NSY	Substitution	p.Ala892Thr
261	myeloid	chr11	119278182	25	0.12	CBL	NSY	Substitution	p.Tyr371Cys
265	myeloid	chr11	119278189	35	0.343	CBL	NSY	Substitution	p.Glu373Asp
267	myeloid	chr11	119278235	34	0.324	CBL	NSY	Substitution	p.Lys389Glu
264	myeloid	chr11	119278238	44	0.068	CBL	NSY	Substitution	p.Asp390Tyr
270	myeloid	chr11	119278257	41	0.122	CBL	NSY	Substitution	p.Cys396Tyr
266	myeloid	chr11	119278272	34	0.118	CBL	NSY	Substitution	p.Cys401Tyr
269	myeloid	chr11	119278281	41	0.073	CBL	NSY	Substitution	p.Cys404Tyr
262	myeloid	chr11	119278520	38	0.158	CBL	NSY	Substitution	p.Gly413Asp
263	myeloid	chr11	119278541	61	0.049	CBL	NSY	Substitution	p.Arg420Gln
268	myeloid	chr11	119278543	36	0.083	CBL	NSY	Substitution	p.Cys421Arg
20	lymphoid	chr1	116536139	84	0.512	CD58	SG	Substitution	p.Arg152X
271	myeloid	chr19	33302246	73	0.041	CEBPA	SG	Substitution	p.Glu57X
91	lymphoid	chr15	92946101	72	0.083	CHD2	NSY	Substitution	p.Pro421Leu
41	lymphoid	chr22	28724976	56	0.071	CHEK2	ESS	Substitution	
42	lymphoid	chr22	28725278	80	0.088	CHEK2	SG	Substitution	p.Arg137X
43	lymphoid	chr22	28725278	67	0.075	CHEK2	SG	Substitution	p.Arg137X
44	lymphoid	chr22	28734402	27	0.259	CHEK2	ESS	Substitution	
45	lymphoid	chr22	28734657	53	0.113	CHEK2	FS	Deletion	
36	lymphoid	chr16	10910260	43	0.116	CIITA	ESS	Substitution	
120	lymphoid	chr4	140392379	53	0.094	CLGN	ESS	Substitution	
107	lymphoid	chr2	237368950	61	0.18	COL6A3	NSY	Substitution	p.Arg1505Cys
108	lymphoid	chr2	237378791	49	0.184	COL6A3	NSY	Substitution	p.Ala781Val

273	myeloid	chr16	3729153	115	0.104	CREBBP	FS	Deletion	
836	myeloid	chr16	3745275	48	0.062	CREBBP	ESS	Substitution	
272	myeloid	chr16	3850317	61	0.049	CREBBP	SG	Substitution	p.Gln260X
111	lymphoid	chr22	36937585	56	0.089	CSF2RB	NSY	Substitution	p.Tyr593Asp
97	lymphoid	chr19	1434830	59	0.102	DAZAP1	FS	Deletion	
75	lymphoid	chr12	31085033	72	0.097	DDX11	NSY	Substitution	p.Pro182Arg
76	lymphoid	chr12	31085033	74	0.176	DDX11	NSY	Substitution	p.Pro182Arg
77	lymphoid	chr12	31091779	86	0.128	DDX11	NSY	Substitution	p.Arg384Trp
78	lymphoid	chr12	31101963	31	0.161	DDX11	NSY	Substitution	p.Arg728His
144	lymphoid	chr7	14338664	58	0.121	DGKB	NSY	Substitution	p.Ile658Thr
150	lymphoid	chr8	13100115	74	0.068	DLC1	NSY	Substitution	p.Ser741Thr
151	lymphoid	chr8	13100148	47	0.191	DLC1	NSY	Substitution	p.Arg730Gln
162	lymphoid	chrX	32343175	63	0.079	DMD	FS	Deletion	
424	myeloid	chr2	25234286	46	0.065	DNMT3A	NSY	Substitution	p.Cys911Tyr
545	myeloid	chr2	25234291	45	0.111	DNMT3A	NSY	Substitution	p.Phe909Leu
459	myeloid	chr2	25234293	42	0.19	DNMT3A	NSY	Substitution	p.Phe909Val
348	myeloid	chr2	25234307	35	0.086	DNMT3A	NSY	Substitution	p.Pro904Leu
403	myeloid	chr2	25234307	38	0.079	DNMT3A	NSY	Substitution	p.Pro904Leu
428	myeloid	chr2	25234307	53	0.113	DNMT3A	NSY	Substitution	p.Pro904Leu
440	myeloid	chr2	25234307	27	0.296	DNMT3A	NSY	Substitution	p.Pro904Leu
519	myeloid	chr2	25234307	46	0.261	DNMT3A	NSY	Substitution	p.Pro904Leu
508	myeloid	chr2	25234343	56	0.071	DNMT3A	SG	Substitution	p.Ser892X
73	myeloid	chr2	25234346	60	0.05	DNMT3A	NSY	Substitution	p.Arg891Gln
295	myeloid	chr2	25234349	67	0.119	DNMT3A	NSY	Substitution	p.Gly890Asp
542	myeloid	chr2	25234358	68	0.103	DNMT3A	NSY	Substitution	p.Arg887Ile
405	myeloid	chr2	25234359	53	0.094	DNMT3A	SG	Substitution	p.Arg887X
521	myeloid	chr2	25234361	54	0.204	DNMT3A	FS	Deletion	
306	myeloid	chr2	25234362	58	0.224	DNMT3A	SG	Substitution	p.Gln886X
356	myeloid	chr2	25234367	54	0.056	DNMT3A	NSY	Substitution	p.Ala884Val
289	myeloid	chr2	25234373	35	0.229	DNMT3A	NSY	Substitution	p.Arg882His
307	myeloid	chr2	25234373	68	0.059	DNMT3A	NSY	Substitution	p.Arg882His

309	myeloid	chr2	25234373	38	0.079	DNMT3A	NSY	Substitution	p.Arg882His
311	myeloid	chr2	25234373	66	0.061	DNMT3A	NSY	Substitution	p.Arg882His
340	myeloid	chr2	25234373	33	0.182	DNMT3A	NSY	Substitution	p.Arg882His
350	myeloid	chr2	25234373	35	0.4	DNMT3A	NSY	Substitution	p.Arg882His
353	myeloid	chr2	25234373	60	0.1	DNMT3A	NSY	Substitution	p.Arg882His
365	myeloid	chr2	25234373	34	0.324	DNMT3A	NSY	Substitution	p.Arg882His
433	myeloid	chr2	25234373	63	0.095	DNMT3A	NSY	Substitution	p.Arg882His
438	myeloid	chr2	25234373	69	0.333	DNMT3A	NSY	Substitution	p.Arg882His
449	myeloid	chr2	25234373	26	0.115	DNMT3A	NSY	Substitution	p.Arg882His
452	myeloid	chr2	25234373	49	0.367	DNMT3A	NSY	Substitution	p.Arg882His
480	myeloid	chr2	25234373	49	0.102	DNMT3A	NSY	Substitution	p.Arg882His
513	myeloid	chr2	25234373	42	0.429	DNMT3A	NSY	Substitution	p.Arg882His
527	myeloid	chr2	25234373	36	0.139	DNMT3A	NSY	Substitution	p.Arg882His
81	myeloid	chr2	25234373	35	0.143	DNMT3A	NSY	Substitution	p.Arg882His
552	myeloid	chr2	25234373	65	0.246	DNMT3A	NSY	Substitution	p.Arg882His
570	myeloid	chr2	25234373	65	0.138	DNMT3A	NSY	Substitution	p.Arg882His
300	myeloid	chr2	25234374	39	0.103	DNMT3A	NSY	Substitution	p.Arg882Cys
316	myeloid	chr2	25234374	45	0.156	DNMT3A	NSY	Substitution	p.Arg882Cys
335	myeloid	chr2	25234374	51	0.059	DNMT3A	NSY	Substitution	p.Arg882Ser
352	myeloid	chr2	25234374	47	0.191	DNMT3A	NSY	Substitution	p.Arg882Cys
394	myeloid	chr2	25234374	41	0.171	DNMT3A	NSY	Substitution	p.Arg882Cys
400	myeloid	chr2	25234374	62	0.065	DNMT3A	NSY	Substitution	p.Arg882Cys
418	myeloid	chr2	25234374	36	0.083	DNMT3A	NSY	Substitution	p.Arg882Cys
445	myeloid	chr2	25234374	66	0.318	DNMT3A	NSY	Substitution	p.Arg882Cys
481	myeloid	chr2	25234374	38	0.132	DNMT3A	NSY	Substitution	p.Arg882Cys
482	myeloid	chr2	25234374	58	0.155	DNMT3A	NSY	Substitution	p.Arg882Cys
490	myeloid	chr2	25234374	45	0.089	DNMT3A	NSY	Substitution	p.Arg882Cys
493	myeloid	chr2	25234374	39	0.077	DNMT3A	NSY	Substitution	p.Arg882Cys
515	myeloid	chr2	25234374	51	0.059	DNMT3A	NSY	Substitution	p.Arg882Cys
567	myeloid	chr2	25234374	51	0.098	DNMT3A	NSY	Substitution	p.Arg882Cys
432	myeloid	chr2	25234380	46	0.13	DNMT3A	NSY	Substitution	p.Met880Val

287	myeloid	chr2	25234383	52	0.135	DNMT3A	FS	Insertion	
494	myeloid	chr2	25234390	42	0.333	DNMT3A	FS	Insertion	
838	myeloid	chr2	25234421	20	0.25	DNMT3A	ESS	Substitution	
839	myeloid	chr2	25234422	25	0.44	DNMT3A	ESS	Substitution	
812	myeloid	chr2	25235706	52	0.096	DNMT3A	ESS	Substitution	
840	myeloid	chr2	25235706	43	0.163	DNMT3A	ESS	Substitution	
492	myeloid	chr2	25235725	81	0.16	DNMT3A	FS	Insertion	
506	myeloid	chr2	25235725	46	0.065	DNMT3A	SG	Substitution	p.Trp860X
337	myeloid	chr2	25235726	60	0.05	DNMT3A	NSY	Substitution	p.Trp860Arg
381	myeloid	chr2	25235726	66	0.136	DNMT3A	NSY	Substitution	p.Trp860Arg
402	myeloid	chr2	25235726	73	0.068	DNMT3A	NSY	Substitution	p.Trp860Arg
426	myeloid	chr2	25235726	60	0.167	DNMT3A	NSY	Substitution	p.Trp860Arg
546	myeloid	chr2	25235726	42	0.238	DNMT3A	NSY	Substitution	p.Trp860Arg
321	myeloid	chr2	25235734	72	0.042	DNMT3A	NSY	Substitution	p.Asp857Val
457	myeloid	chr2	25235751	76	0.105	DNMT3A	FS	Deletion	
441	myeloid	chr2	25235756	68	0.176	DNMT3A	NSY	Substitution	p.Val850Phe
541	myeloid	chr2	25235767	53	0.057	DNMT3A	NSY	Substitution	p.Gln846Arg
538	myeloid	chr2	25235768	115	0.035	DNMT3A	SG	Substitution	p.Gln846X
568	myeloid	chr2	25235771	74	0.095	DNMT3A	FS	Insertion	
310	myeloid	chr2	25235782	47	0.106	DNMT3A	NSY	Substitution	p.Lys841Thr
412	myeloid	chr2	25235792	73	0.041	DNMT3A	NSY	Substitution	p.Asn838Asp
182	myeloid	chr2	25236965	66	0.318	DNMT3A	SG	Substitution	p.Glu817X
444	myeloid	chr2	25237000	31	0.097	DNMT3A	SG	Substitution	p.Leu805X
283	myeloid	chr2	25239143	42	0.071	DNMT3A	NSY	Substitution	p.Pro799Ala
359	myeloid	chr2	25239145	36	0.194	DNMT3A	NSY	Substitution	p.Leu798Arg
566	myeloid	chr2	25239149	38	0.105	DNMT3A	NSY	Substitution	p.Asn797Asp
366	myeloid	chr2	25239151	60	0.133	DNMT3A	NSY	Substitution	p.Gly796Val
391	myeloid	chr2	25239154	39	0.256	DNMT3A	SG	Substitution	p.Trp795X
544	myeloid	chr2	25239154	44	0.159	DNMT3A	SG	Substitution	p.Trp795X
477	myeloid	chr2	25239177	50	0.08	DNMT3A	FS	Deletion	
551	myeloid	chr2	25239178	27	0.111	DNMT3A	NSY	Substitution	p.Ala787Val

303	myeloid	chr2	25239181	37	0.108	DNMT3A	NSY	Substitution	p.Ser786Leu
282	myeloid	chr2	25239187	34	0.088	DNMT3A	FS	Deletion	
384	myeloid	chr2	25239199	45	0.067	DNMT3A	NSY	Substitution	p.Ile780Thr
435	myeloid	chr2	25239206	38	0.079	DNMT3A	NSY	Substitution	p.Val778Met
414	myeloid	chr2	25239208	48	0.062	DNMT3A	NSY	Substitution	p.Pro777Leu
841	myeloid	chr2	25239217	48	0.062	DNMT3A	ESS	Substitution	
842	myeloid	chr2	25240300	34	0.176	DNMT3A	ESS	Substitution	
843	myeloid	chr2	25240301	33	0.152	DNMT3A	ESS	Substitution	
470	myeloid	chr2	25240307	46	0.087	DNMT3A	NSY	Substitution	p.Leu773Ile
363	myeloid	chr2	25240310	31	0.129	DNMT3A	NSY	Substitution	p.Phe772Ile
299	myeloid	chr2	25240312	52	0.288	DNMT3A	NSY	Substitution	p.Arg771Gln
317	myeloid	chr2	25240312	53	0.208	DNMT3A	NSY	Substitution	p.Arg771Gln
398	myeloid	chr2	25240312	29	0.103	DNMT3A	NSY	Substitution	p.Arg771Gln
572	myeloid	chr2	25240312	26	0.192	DNMT3A	NSY	Substitution	p.Arg771Gln
278	myeloid	chr2	25240313	37	0.081	DNMT3A	NSY	Substitution	p.Arg771Gly
447	myeloid	chr2	25240313	46	0.109	DNMT3A	SG	Substitution	p.Arg771X
491	myeloid	chr2	25240313	51	0.235	DNMT3A	SG	Substitution	p.Arg771X
525	myeloid	chr2	25240313	35	0.143	DNMT3A	SG	Substitution	p.Arg771X
533	myeloid	chr2	25240313	31	0.097	DNMT3A	SG	Substitution	p.Arg771X
499	myeloid	chr2	25240315	52	0.077	DNMT3A	NSY	Substitution	p.Ser770Leu
504	myeloid	chr2	25240315	29	0.241	DNMT3A	NSY	Substitution	p.Ser770Leu
530	myeloid	chr2	25240315	47	0.085	DNMT3A	FS	Insertion	
532	myeloid	chr2	25240315	41	0.171	DNMT3A	NSY	Substitution	p.Ser770Leu
562	myeloid	chr2	25240315	48	0.104	DNMT3A	NSY	Substitution	p.Ser770Leu
563	myeloid	chr2	25240315	32	0.125	DNMT3A	NSY	Substitution	p.Ser770Trp
322	myeloid	chr2	25240326	45	0.089	DNMT3A	FS	Deletion	
509	myeloid	chr2	25240335	68	0.074	DNMT3A	FS	Deletion	
274	myeloid	chr2	25240360	54	0.056	DNMT3A	NSY	Substitution	p.Phe755Ser
293	myeloid	chr2	25240360	44	0.114	DNMT3A	NSY	Substitution	p.Phe755Ser
325	myeloid	chr2	25240360	63	0.063	DNMT3A	NSY	Substitution	p.Phe755Ser
326	myeloid	chr2	25240360	62	0.081	DNMT3A	NSY	Substitution	p.Phe755Ser

547	myeloid	chr2	25240360	57	0.228	DNMT3A	NSY	Substitution	p.Phe755Ser
378	myeloid	chr2	25240367	49	0.061	DNMT3A	NSY	Substitution	p.Trp753Arg
301	myeloid	chr2	25240369	80	0.138	DNMT3A	NSY	Substitution	p.Phe752Cys
560	myeloid	chr2	25240373	79	0.316	DNMT3A	NSY	Substitution	p.Phe751Leu
500	myeloid	chr2	25240376	72	0.056	DNMT3A	NSY	Substitution	p.Pro750Ser
534	myeloid	chr2	25240378	76	0.039	DNMT3A	NSY	Substitution	p.Arg749His
318	myeloid	chr2	25240379	84	0.083	DNMT3A	NSY	Substitution	p.Arg749Cys
344	myeloid	chr2	25240379	69	0.217	DNMT3A	NSY	Substitution	p.Arg749Cys
461	myeloid	chr2	25240379	57	0.053	DNMT3A	NSY	Substitution	p.Arg749Cys
497	myeloid	chr2	25240379	59	0.254	DNMT3A	NSY	Substitution	p.Arg749Cys
550	myeloid	chr2	25240379	84	0.071	DNMT3A	NSY	Substitution	p.Arg749Cys
557	myeloid	chr2	25240379	57	0.281	DNMT3A	NSY	Substitution	p.Arg749Gly
302	myeloid	chr2	25240392	47	0.106	DNMT3A	FS	Insertion	
501	myeloid	chr2	25240394	67	0.06	DNMT3A	FS	Deletion	
304	myeloid	chr2	25240396	108	0.065	DNMT3A	NSY	Substitution	p.Pro743Arg
404	myeloid	chr2	25240402	57	0.105	DNMT3A	FS	Insertion	
471	myeloid	chr2	25240414	44	0.091	DNMT3A	NSY	Substitution	p.Leu737Arg
338	myeloid	chr2	25240417	55	0.145	DNMT3A	NSY	Substitution	p.Arg736His
376	myeloid	chr2	25240417	56	0.054	DNMT3A	NSY	Substitution	p.Arg736Leu
383	myeloid	chr2	25240417	51	0.314	DNMT3A	NSY	Substitution	p.Arg736His
479	myeloid	chr2	25240417	59	0.051	DNMT3A	NSY	Substitution	p.Arg736His
489	myeloid	chr2	25240417	46	0.087	DNMT3A	NSY	Substitution	p.Arg736His
558	myeloid	chr2	25240417	34	0.265	DNMT3A	NSY	Substitution	p.Arg736His
569	myeloid	chr2	25240417	74	0.041	DNMT3A	NSY	Substitution	p.Arg736His
312	myeloid	chr2	25240418	54	0.167	DNMT3A	NSY	Substitution	p.Arg736Cys
361	myeloid	chr2	25240418	40	0.075	DNMT3A	NSY	Substitution	p.Arg736Cys
367	myeloid	chr2	25240418	49	0.184	DNMT3A	NSY	Substitution	p.Arg736Cys
409	myeloid	chr2	25240418	45	0.267	DNMT3A	NSY	Substitution	p.Arg736Cys
39	myeloid	chr2	25240418	56	0.125	DNMT3A	NSY	Substitution	p.Arg736Cys
329	myeloid	chr2	25240420	51	0.196	DNMT3A	NSY	Substitution	p.Tyr735Cys
434	myeloid	chr2	25240420	33	0.212	DNMT3A	NSY	Substitution	p.Tyr735Cys

520	myeloid	chr2	25240420	47	0.255	DNMT3A	NSY	Substitution	p.Tyr735Cys
336	myeloid	chr2	25240422	45	0.089	DNMT3A	FS	Deletion	
451	myeloid	chr2	25240422	75	0.08	DNMT3A	NSY	Substitution	p.Phe734Leu
467	myeloid	chr2	25240422	38	0.132	DNMT3A	NSY	Substitution	p.Phe734Leu
385	myeloid	chr2	25240424	63	0.111	DNMT3A	NSY	Substitution	p.Phe734Val
572	myeloid	chr2	25240424	32	0.219	DNMT3A	FS	Insertion	
294	myeloid	chr2	25240426	32	0.125	DNMT3A	NSY	Substitution	p.Glu733Ala
375	myeloid	chr2	25240426	45	0.133	DNMT3A	FS	Deletion	
297	myeloid	chr2	25240427	54	0.111	DNMT3A	NSY	Substitution	p.Glu733Gln
324	myeloid	chr2	25240430	51	0.333	DNMT3A	FS	Deletion	
476	myeloid	chr2	25240433	43	0.14	DNMT3A	NSY	Substitution	p.Phe731Val
477	myeloid	chr2	25240433	58	0.052	DNMT3A	NSY	Substitution	p.Phe731Ile
286	myeloid	chr2	25240438	26	0.154	DNMT3A	NSY	Substitution	p.Arg729Gln
468	myeloid	chr2	25240438	43	0.07	DNMT3A	NSY	Substitution	p.Arg729Gln
507	myeloid	chr2	25240438	37	0.081	DNMT3A	NSY	Substitution	p.Arg729Gln
429	myeloid	chr2	25240439	27	0.111	DNMT3A	NSY	Substitution	p.Arg729Trp
436	myeloid	chr2	25240441	49	0.061	DNMT3A	NSY	Substitution	p.Gly728Asp
844	myeloid	chr2	25240639	36	0.139	DNMT3A	ESS	Substitution	
845	myeloid	chr2	25240639	38	0.079	DNMT3A	ESS	Substitution	
382	myeloid	chr2	25240643	28	0.179	DNMT3A	FS	Deletion	
423	myeloid	chr2	25240647	38	0.211	DNMT3A	FS	Deletion	
347	myeloid	chr2	25240648	35	0.114	DNMT3A	NSY	Substitution	p.Gly722Asp
455	myeloid	chr2	25240667	57	0.07	DNMT3A	NSY	Substitution	p.Val716Phe
305	myeloid	chr2	25240672	48	0.125	DNMT3A	NSY	Substitution	p.Ser714Cys
524	myeloid	chr2	25240672	60	0.117	DNMT3A	NSY	Substitution	p.Ser714Cys
370	myeloid	chr2	25240684	41	0.22	DNMT3A	FS	Insertion	
397	myeloid	chr2	25240692	42	0.071	DNMT3A	FS	Deletion	
540	myeloid	chr2	25240692	53	0.113	DNMT3A	FS	Deletion	
277	myeloid	chr2	25240697	53	0.057	DNMT3A	NSY	Substitution	p.Gly706Trp
466	myeloid	chr2	25240697	39	0.077	DNMT3A	NSY	Substitution	p.Gly706Trp
548	myeloid	chr2	25240697	50	0.08	DNMT3A	NSY	Substitution	p.Gly706Arg

284	myeloid	chr2	25240712	33	0.212	DNMT3A	NSY	Substitution	p.Phe701Leu
349	myeloid	chr2	25240719	34	0.265	DNMT3A	SG	Substitution	p.Trp698X
374	myeloid	chr2	25240726	25	0.2	DNMT3A	FS	Deletion	
510	myeloid	chr2	25241572	31	0.194	DNMT3A	FS	Insertion	
485	myeloid	chr2	25241581	32	0.188	DNMT3A	NSY	Substitution	p.Arg688His
392	myeloid	chr2	25241582	33	0.091	DNMT3A	NSY	Substitution	p.Arg688Cys
308	myeloid	chr2	25241595	43	0.116	DNMT3A	SG	Substitution	p.Tyr683X
357	myeloid	chr2	25241596	54	0.056	DNMT3A	FS	Deletion	
460	myeloid	chr2	25241617	41	0.098	DNMT3A	NSY	Substitution	p.Arg676Gln
355	myeloid	chr2	25241618	51	0.157	DNMT3A	NSY	Substitution	p.Arg676Trp
529	myeloid	chr2	25241618	49	0.061	DNMT3A	NSY	Substitution	p.Arg676Trp
330	myeloid	chr2	25241621	46	0.065	DNMT3A	NSY	Substitution	p.Val675Met
450	myeloid	chr2	25241636	46	0.065	DNMT3A	FS	Insertion	
110	myeloid	chr2	25241663	40	0.175	DNMT3A	FS	Insertion	
415	myeloid	chr2	25241664	40	0.1	DNMT3A	SG	Substitution	p.Tyr660X
82	myeloid	chr2	25241665	62	0.177	DNMT3A	NSY	Substitution	p.Tyr660Cys
495	myeloid	chr2	25241665	41	0.098	DNMT3A	NSY	Substitution	p.Tyr660Cys
292	myeloid	chr2	25241675	47	0.064	DNMT3A	NSY	Substitution	p.Val657Met
379	myeloid	chr2	25241675	30	0.1	DNMT3A	NSY	Substitution	p.Val657Met
421	myeloid	chr2	25241675	37	0.081	DNMT3A	NSY	Substitution	p.Val657Met
528	myeloid	chr2	25241675	82	0.037	DNMT3A	NSY	Substitution	p.Val657Leu
464	myeloid	chr2	25241695	40	0.2	DNMT3A	NSY	Substitution	p.Leu650Pro
505	myeloid	chr2	25241695	34	0.088	DNMT3A	NSY	Substitution	p.Leu650Gln
846	myeloid	chr2	25241708	25	0.4	DNMT3A	ESS	Substitution	
847	myeloid	chr2	25243897	49	0.082	DNMT3A	ESS	Substitution	
488	myeloid	chr2	25243915	25	0.2	DNMT3A	FS	Deletion	
346	myeloid	chr2	25243924	53	0.057	DNMT3A	NSY	Substitution	p.Leu637Pro
413	myeloid	chr2	25243927	44	0.136	DNMT3A	NSY	Substitution	p.Val636Gly
554	myeloid	chr2	25243927	51	0.059	DNMT3A	NSY	Substitution	p.Val636Gly
327	myeloid	chr2	25243928	58	0.103	DNMT3A	NSY	Substitution	p.Val636Met
345	myeloid	chr2	25243928	57	0.07	DNMT3A	NSY	Substitution	p.Val636Met

320	myeloid	chr2	25243930	55	0.291	DNMT3A	NSY	Substitution	p.Arg635Gln
458	myeloid	chr2	25243930	35	0.229	DNMT3A	NSY	Substitution	p.Arg635Gln
531	myeloid	chr2	25243930	44	0.136	DNMT3A	NSY	Substitution	p.Arg635Pro
275	myeloid	chr2	25243931	51	0.059	DNMT3A	NSY	Substitution	p.Arg635Trp
371	myeloid	chr2	25243931	59	0.186	DNMT3A	NSY	Substitution	p.Arg635Trp
373	myeloid	chr2	25243931	38	0.158	DNMT3A	NSY	Substitution	p.Arg635Trp
396	myeloid	chr2	25243931	43	0.07	DNMT3A	NSY	Substitution	p.Arg635Trp
427	myeloid	chr2	25243931	55	0.127	DNMT3A	NSY	Substitution	p.Arg635Trp
448	myeloid	chr2	25243931	76	0.039	DNMT3A	NSY	Substitution	p.Arg635Trp
514	myeloid	chr2	25243931	71	0.197	DNMT3A	NSY	Substitution	p.Arg635Trp
518	myeloid	chr2	25243931	55	0.055	DNMT3A	NSY	Substitution	p.Arg635Trp
362	myeloid	chr2	25243933	66	0.091	DNMT3A	NSY	Substitution	p.Ile634Ser
564	myeloid	chr2	25243941	49	0.082	DNMT3A	NSY	Substitution	p.Arg631Ser
279	myeloid	chr2	25243945	47	0.234	DNMT3A	FS	Insertion	
280	myeloid	chr2	25244160	24	0.125	DNMT3A	SG	Substitution	p.Glu616X
454	myeloid	chr2	25244160	43	0.116	DNMT3A	SG	Substitution	p.Glu616X
331	myeloid	chr2	25244193	30	0.1	DNMT3A	NSY	Substitution	p.Leu605Ile
372	myeloid	chr2	25244203	53	0.075	DNMT3A	SG	Substitution	p.Trp601X
425	myeloid	chr2	25244214	45	0.156	DNMT3A	SG	Substitution	p.Arg598X
430	myeloid	chr2	25244214	43	0.07	DNMT3A	SG	Substitution	p.Arg598X
565	myeloid	chr2	25244214	50	0.12	DNMT3A	SG	Substitution	p.Arg598X
388	myeloid	chr2	25244217	39	0.077	DNMT3A	NSY	Substitution	p.Arg597Trp
380	myeloid	chr2	25244222	54	0.093	DNMT3A	NSY	Substitution	p.Leu595Pro
276	myeloid	chr2	25244244	41	0.098	DNMT3A	FS	Deletion	
368	myeloid	chr2	25244244	40	0.1	DNMT3A	FS	Deletion	
469	myeloid	chr2	25244259	58	0.052	DNMT3A	NSY	Substitution	p.Cys583Ser
339	myeloid	chr2	25244265	80	0.162	DNMT3A	NSY	Substitution	p.Trp581Arg
351	myeloid	chr2	25244265	52	0.058	DNMT3A	NSY	Substitution	p.Trp581Arg
393	myeloid	chr2	25244270	54	0.222	DNMT3A	FS	Deletion	
496	myeloid	chr2	25244279	75	0.04	DNMT3A	NSY	Substitution	p.Ile576Asn
465	myeloid	chr2	25244289	48	0.062	DNMT3A	SG	Substitution	p.Gln573X

386	myeloid	chr2	25244294	36	0.194	DNMT3A	FS	Deletion	
517	myeloid	chr2	25244294	38	0.079	DNMT3A	FS	Deletion	
472	myeloid	chr2	25244319	39	0.128	DNMT3A	NSY	Substitution	p.Val563Met
848	myeloid	chr2	25244339	26	0.115	DNMT3A	ESS	Substitution	
849	myeloid	chr2	25244340	32	0.094	DNMT3A	ESS	Substitution	
850	myeloid	chr2	25244539	40	0.1	DNMT3A	ESS	Substitution	
330	myeloid	chr2	25244539	30	0.167	DNMT3A	ESS	Substitution	
851	myeloid	chr2	25244539	32	0.125	DNMT3A	ESS	Substitution	
522	myeloid	chr2	25244550	36	0.278	DNMT3A	FS	Deletion	
463	myeloid	chr2	25244551	29	0.241	DNMT3A	FS	Deletion	
539	myeloid	chr2	25244560	36	0.083	DNMT3A	NSY	Substitution	p.Cys549Trp
334	myeloid	chr2	25244579	38	0.079	DNMT3A	NSY	Substitution	p.Gly543Val
456	myeloid	chr2	25244580	31	0.29	DNMT3A	NSY	Substitution	p.Gly543Cys
302	myeloid	chr2	25244612	29	0.103	DNMT3A	NSY	Substitution	p.Gly532Asp
437	myeloid	chr2	25244647	39	0.179	DNMT3A	SG	Substitution	p.Cys520X
852	myeloid	chr2	25244654	29	0.103	DNMT3A	ESS	Substitution	
853	myeloid	chr2	25244654	26	0.115	DNMT3A	ESS	Substitution	
854	myeloid	chr2	25244654	27	0.111	DNMT3A	ESS	Substitution	
855	myeloid	chr2	25245252	43	0.116	DNMT3A	ESS	Substitution	
498	myeloid	chr2	25245259	48	0.104	DNMT3A	FS	Deletion	
313	myeloid	chr2	25245294	67	0.045	DNMT3A	SG	Substitution	p.Glu505X
453	myeloid	chr2	25245298	67	0.149	DNMT3A	FS	Deletion	
343	myeloid	chr2	25245299	36	0.083	DNMT3A	FS	Insertion	
332	myeloid	chr2	25245316	37	0.081	DNMT3A	NSY	Substitution	p.Cys497Trp
856	myeloid	chr2	25246018	55	0.127	DNMT3A	ESS	Deletion	
857	myeloid	chr2	25246018	43	0.116	DNMT3A	ESS	Deletion	
439	myeloid	chr2	25246019	20	0.15	DNMT3A	ESS	Substitution	
858	myeloid	chr2	25246066	27	0.185	DNMT3A	ESS	Substitution	
859	myeloid	chr2	25246159	56	0.071	DNMT3A	ESS	Substitution	
860	myeloid	chr2	25246159	37	0.081	DNMT3A	ESS	Substitution	
511	myeloid	chr2	25246193	39	0.256	DNMT3A	FS	Deletion	

486	myeloid	chr2	25246221	56	0.071	DNMT3A	FS	Deletion	
360	myeloid	chr2	25246240	72	0.042	DNMT3A	FS	Insertion	
342	myeloid	chr2	25246245	86	0.035	DNMT3A	SG	Substitution	p.Tyr448X
71	myeloid	chr2	25246259	83	0.06	DNMT3A	FS	Deletion	
296	myeloid	chr2	25246270	43	0.07	DNMT3A	SG	Substitution	p.Trp440X
503	myeloid	chr2	25246270	75	0.08	DNMT3A	SG	Substitution	p.Trp440X
288	myeloid	chr2	25246276	60	0.117	DNMT3A	FS	Deletion	
290	myeloid	chr2	25246620	22	0.136	DNMT3A	SG	Substitution	p.Glu427X
446	myeloid	chr2	25246620	29	0.172	DNMT3A	FS	Deletion	
185	myeloid	chr2	25246635	36	0.25	DNMT3A	FS	Deletion	
536	myeloid	chr2	25246656	46	0.065	DNMT3A	SG	Substitution	p.Gln415X
449	myeloid	chr2	25246660	55	0.127	DNMT3A	FS	Deletion	
553	myeloid	chr2	25246660	32	0.219	DNMT3A	FS	Deletion	
333	myeloid	chr2	25246672	37	0.108	DNMT3A	SG	Substitution	p.Trp409X
406	myeloid	chr2	25246677	52	0.096	DNMT3A	SG	Substitution	p.Glu408X
420	myeloid	chr2	25246695	44	0.091	DNMT3A	SG	Substitution	p.Gln402X
559	myeloid	chr2	25246701	51	0.078	DNMT3A	SG	Substitution	p.Glu400X
526	myeloid	chr2	25246704	64	0.172	DNMT3A	FS	Insertion	
314	myeloid	chr2	25246713	76	0.039	DNMT3A	FS	Insertion	
285	myeloid	chr2	25246734	48	0.062	DNMT3A	NSY	Substitution	p.Asp389Asn
337	myeloid	chr2	25246747	44	0.182	DNMT3A	FS	Deletion	
364	myeloid	chr2	25246761	39	0.077	DNMT3A	NSY	Substitution	p.Ala380Ser
861	myeloid	chr2	25246778	37	0.243	DNMT3A	ESS	Substitution	
419	myeloid	chr2	25247071	36	0.278	DNMT3A	NSY	Substitution	p.Ala368Thr
390	myeloid	chr2	25247076	23	0.261	DNMT3A	NSY	Substitution	p.Arg366His
410	myeloid	chr2	25247079	30	0.133	DNMT3A	NSY	Substitution	p.Tyr365Cys
483	myeloid	chr2	25247103	35	0.114	DNMT3A	FS	Deletion	
543	myeloid	chr2	25247107	42	0.071	DNMT3A	SG	Substitution	p.Gln356X
416	myeloid	chr2	25247123	47	0.085	DNMT3A	FS	Insertion	
369	myeloid	chr2	25247133	21	0.143	DNMT3A	NSY	Substitution	p.Leu347Gln
561	myeloid	chr2	25247133	39	0.179	DNMT3A	NSY	Substitution	p.Leu347Pro

411	myeloid	chr2	25247137	26	0.115	DNMT3A	NSY	Substitution	p.Pro346Ser
431	myeloid	chr2	25247137	45	0.089	DNMT3A	FS	Deletion	
474	myeloid	chr2	25247595	25	0.12	DNMT3A	SG	Substitution	p.Ser337X
487	myeloid	chr2	25247606	41	0.244	DNMT3A	FS	Deletion	
387	myeloid	chr2	25247611	39	0.077	DNMT3A	NSY	Substitution	p.Gly332Arg
395	myeloid	chr2	25247615	43	0.233	DNMT3A	SG	Substitution	p.Trp330X
502	myeloid	chr2	25247615	72	0.097	DNMT3A	SG	Substitution	p.Trp330X
389	myeloid	chr2	25247628	42	0.119	DNMT3A	NSY	Substitution	p.Arg326His
408	myeloid	chr2	25247628	46	0.217	DNMT3A	NSY	Substitution	p.Arg326His
417	myeloid	chr2	25247628	41	0.195	DNMT3A	NSY	Substitution	p.Arg326His
475	myeloid	chr2	25247629	53	0.075	DNMT3A	NSY	Substitution	p.Arg326Cys
478	myeloid	chr2	25247629	46	0.152	DNMT3A	NSY	Substitution	p.Arg326Cys
523	myeloid	chr2	25247629	69	0.087	DNMT3A	NSY	Substitution	p.Arg326Cys
298	myeloid	chr2	25247647	47	0.106	DNMT3A	SG	Substitution	p.Arg320X
328	myeloid	chr2	25247647	71	0.042	DNMT3A	SG	Substitution	p.Arg320X
442	myeloid	chr2	25247647	48	0.5	DNMT3A	SG	Substitution	p.Arg320X
549	myeloid	chr2	25247647	63	0.063	DNMT3A	SG	Substitution	p.Arg320X
439	myeloid	chr2	25247663	64	0.047	DNMT3A	SG	Substitution	p.Trp314X
422	myeloid	chr2	25247666	60	0.05	DNMT3A	SG	Substitution	p.Trp313X
401	myeloid	chr2	25247676	55	0.127	DNMT3A	NSY	Substitution	p.Ile310Thr
407	myeloid	chr2	25247679	65	0.046	DNMT3A	NSY	Substitution	p.Arg309His
443	myeloid	chr2	25247682	73	0.041	DNMT3A	NSY	Substitution	p.Gly308Asp
399	myeloid	chr2	25247683	74	0.095	DNMT3A	NSY	Substitution	p.Gly308Arg
555	myeloid	chr2	25247685	59	0.068	DNMT3A	NSY	Substitution	p.Pro307Arg
281	myeloid	chr2	25247686	72	0.083	DNMT3A	NSY	Substitution	p.Pro307Ser
354	myeloid	chr2	25247695	58	0.069	DNMT3A	NSY	Substitution	p.Ser304Pro
516	myeloid	chr2	25247698	67	0.045	DNMT3A	NSY	Substitution	p.Phe303Leu
358	myeloid	chr2	25247710	31	0.161	DNMT3A	FS	Deletion	
291	myeloid	chr2	25247712	53	0.057	DNMT3A	NSY	Substitution	p.Gly298Glu
315	myeloid	chr2	25247713	51	0.118	DNMT3A	NSY	Substitution	p.Gly298Arg
377	myeloid	chr2	25247713	54	0.056	DNMT3A	NSY	Substitution	p.Gly298Arg

319	myeloid	chr2	25247719	40	0.225	DNMT3A	NSY	Substitution	p.Val296Met
473	myeloid	chr2	25247719	50	0.1	DNMT3A	NSY	Substitution	p.Val296Leu
546	myeloid	chr2	25247719	38	0.105	DNMT3A	NSY	Substitution	p.Val296Met
556	myeloid	chr2	25247719	49	0.143	DNMT3A	NSY	Substitution	p.Val296Leu
484	myeloid	chr2	25247721	66	0.121	DNMT3A	NSY	Substitution	p.Leu295Gln
462	myeloid	chr2	25247724	46	0.261	DNMT3A	FS	Deletion	
862	myeloid	chr2	25248036	30	0.2	DNMT3A	ESS	Substitution	
863	myeloid	chr2	25248036	27	0.407	DNMT3A	ESS	Substitution	
524	myeloid	chr2	25248045	45	0.067	DNMT3A	SG	Substitution	p.Glu283X
571	myeloid	chr2	25248063	48	0.104	DNMT3A	FS	Deletion	
537	myeloid	chr2	25248093	41	0.146	DNMT3A	FS	Deletion	
535	myeloid	chr2	25248099	43	0.07	DNMT3A	FS	Deletion	
341	myeloid	chr2	25274999	64	0.047	DNMT3A	FS	Deletion	
323	myeloid	chr2	25275528	63	0.063	DNMT3A	FS	Deletion	
512	myeloid	chr2	25282561	65	0.123	DNMT3A	FS	Deletion	
130	lymphoid	chr6	335142	105	0.105	DUSP22	NSY	Substitution	p.Ala56Val
131	lymphoid	chr6	335142	64	0.141	DUSP22	NSY	Substitution	p.Ala56Val
132	lymphoid	chr6	335142	86	0.151	DUSP22	NSY	Substitution	p.Ala56Val
133	lymphoid	chr6	335142	66	0.182	DUSP22	NSY	Substitution	p.Ala56Val
134	lymphoid	chr6	335142	49	0.184	DUSP22	NSY	Substitution	p.Ala56Val
135	lymphoid	chr6	345906	57	0.193	DUSP22	NSY	Substitution	p.Gly81Ser
136	lymphoid	chr6	348125	41	0.122	DUSP22	NSY	Substitution	p.Val96Met
137	lymphoid	chr6	348155	68	0.147	DUSP22	NSY	Substitution	p.Val106Ile
152	lymphoid	chr8	23444562	42	0.167	ENTPD4	NSY	Substitution	p.Ile153Val
574	myeloid	chr22	41117348	58	0.052	EP300	SG	Substitution	p.Arg86X
323	myeloid	chr22	41117696	54	0.056	EP300	SG	Substitution	p.Arg202X
573	myeloid	chr22	41117696	88	0.034	EP300	SG	Substitution	p.Arg202X
106	lymphoid	chr2	211383888	57	0.105	ERBB4	NSY	Substitution	p.Lys1218Asn
74	lymphoid	chr11	128490499	65	0.185	ETS1	NSY	Substitution	p.Ser98Cys
575	myeloid	chr12	11853525	67	0.045	ETV6	SG	Substitution	p.Gln143X
576	myeloid	chr7	148814069	53	0.057	EZH2	NSY	Substitution	p.Ala581Thr

138	lymphoid	chr6	35455919	72	0.111	FANCE	NSY	Substitution	p.Arg141Gly
23	lymphoid	chr10	89014137	106	0.057	FAS	FS	Insertion	
42	lymphoid	chr4	186611757	48	0.083	FAT1	SG	Substitution	p.Leu3161X
48	lymphoid	chr4	186617114	66	0.045	FAT1	FS	Deletion	
121	lymphoid	chr4	186617993	71	0.113	FAT1	NSY	Substitution	p.Glu2865Lys
122	lymphoid	chr4	186618051	35	0.143	FAT1	NSY	Substitution	p.Met2845Ile
49	lymphoid	chr4	186618088	40	0.275	FAT1	FS	Deletion	
123	lymphoid	chr4	186620716	62	0.113	FAT1	NSY	Substitution	p.Gly1957Asp
124	lymphoid	chr4	186628319	30	0.2	FAT1	NSY	Substitution	p.Val1549Met
125	lymphoid	chr4	186707666	83	0.133	FAT1	NSY	Substitution	p.Pro721Leu
126	lymphoid	chr4	186708182	55	0.109	FAT1	NSY	Substitution	p.Arg549Gln
50	lymphoid	chr4	186709434	57	0.053	FAT1	SG	Substitution	p.Arg132X
129	lymphoid	chr5	151534499	45	0.111	FAT2	NSY	Substitution	p.Phe3113Val
116	lymphoid	chr4	125320202	66	0.076	FAT4	NSY	Substitution	p.Ser1264Phe
117	lymphoid	chr4	125416590	50	0.16	FAT4	NSY	Substitution	p.Arg2329Leu
118	lymphoid	chr4	125450178	42	0.167	FAT4	FS	Deletion	
119	lymphoid	chr4	125490659	62	0.113	FAT4	NSY	Substitution	p.Pro4615Ala
577	myeloid	chr13	28028192	38	0.395	FLT3	NSY	Substitution	p.Ala680Val
578	myeloid	chr13	28033902	53	0.528	FLT3	NSY	Substitution	p.Val643Ile
34	lymphoid	chr13	40666143	61	0.066	FOXO1	NSY	Substitution	p.Thr24Ala
35	lymphoid	chr13	40666148	70	0.057	FOXO1	NSY	Substitution	p.Ser22Trp
588	myeloid	chr1	1815788	69	0.072	GNB1	NSY	Substitution	p.Lys57Asn
579	myeloid	chr1	1815790	31	0.097	GNB1	NSY	Substitution	p.Lys57Glu
580	myeloid	chr1	1815790	57	0.316	GNB1	NSY	Substitution	p.Lys57Glu
581	myeloid	chr1	1815790	53	0.132	GNB1	NSY	Substitution	p.Lys57Glu
582	myeloid	chr1	1815790	45	0.244	GNB1	NSY	Substitution	p.Lys57Glu
583	myeloid	chr1	1815790	49	0.327	GNB1	NSY	Substitution	p.Lys57Glu
584	myeloid	chr1	1815790	23	0.348	GNB1	NSY	Substitution	p.Lys57Glu
585	myeloid	chr1	1815790	58	0.121	GNB1	NSY	Substitution	p.Lys57Glu
586	myeloid	chr1	1815790	68	0.265	GNB1	NSY	Substitution	p.Lys57Glu
587	myeloid	chr1	1815790	39	0.077	GNB1	NSY	Substitution	p.Lys57Glu

589	myeloid	chr1	1815790	52	0.115	GNB1	NSY	Substitution	p.Lys57Glu
590	myeloid	chr1	1815790	49	0.122	GNB1	NSY	Substitution	p.Lys57Glu
112	lymphoid	chr3	7578807	40	0.175	GRM7	NSY	Substitution	p.Thr634Met
115	lymphoid	chr4	82373601	67	0.179	HNRNPD	NSY	Substitution	p.Gln26His
12	lymphoid	chr1	23559237	47	0.064	ID3	NSY	Substitution	p.Leu64Phe
344	myeloid	chr15	90088702	59	0.102	IDH2	NSY	Substitution	p.Arg140Gln
82	myeloid	chr15	90088702	64	0.125	IDH2	NSY	Substitution	p.Arg140Gln
203	myeloid	chr15	90088702	55	0.309	IDH2	NSY	Substitution	p.Arg140Gln
591	myeloid	chr15	90088702	47	0.213	IDH2	NSY	Substitution	p.Arg140Gln
110	lymphoid	chr22	22895444	44	0.114	IGLL5	NSY	Substitution	p.Ala132Asp
592	myeloid	chr7	50376619	62	0.048	IKZF1	SG	Substitution	p.Arg83X
69	lymphoid	chr1	234609230	75	0.093	IRF2BP2	NSY	Substitution	p.Lys89Gln
68	lymphoid	chr1	226736468	48	0.104	ITPKB	NSY	Substitution	p.Arg331Ser
593	myeloid	chr9	5073770	79	0.063	JAK2	NSY	Substitution	p.Val617Phe
594	myeloid	chr9	5073770	54	0.056	JAK2	NSY	Substitution	p.Val617Phe
595	myeloid	chr9	5073770	51	0.118	JAK2	NSY	Substitution	p.Val617Phe
596	myeloid	chr9	5073770	52	0.173	JAK2	NSY	Substitution	p.Val617Phe
597	myeloid	chr9	5073770	41	0.073	JAK2	NSY	Substitution	p.Val617Phe
598	myeloid	chr9	5073770	56	0.071	JAK2	NSY	Substitution	p.Val617Phe
599	myeloid	chr9	5073770	66	0.061	JAK2	NSY	Substitution	p.Val617Phe
600	myeloid	chr9	5073770	31	0.452	JAK2	NSY	Substitution	p.Val617Phe
601	myeloid	chr9	5073770	62	0.274	JAK2	NSY	Substitution	p.Val617Phe
602	myeloid	chr9	5073770	46	0.217	JAK2	NSY	Substitution	p.Val617Phe
603	myeloid	chr9	5073770	62	0.048	JAK2	NSY	Substitution	p.Val617Phe
606	myeloid	chrX	45063449	40	0.15	KDM6A	SG	Substitution	p.Arg571X
604	myeloid	chrX	45069783	54	0.278	KDM6A	SG	Substitution	p.Gln762X
605	myeloid	chrX	45089911	73	0.315	KDM6A	SG	Substitution	p.Trp1291X
607	myeloid	chr4	54733155	55	0.109	KIT	NSY	Substitution	p.Asp816Val
96	lymphoid	chr18	32680235	94	0.085	KLHL14	NSY	Substitution	p.Arg508Cys
59	lymphoid	chr1	6602153	82	0.183	KLHL21	NSY	Substitution	p.Glu222Val
60	lymphoid	chr1	6602223	127	0.063	KLHL21	NSY	Substitution	p.Tyr199His

159	lymphoid	chr9	21334771	59	0.085	KLHL9	NSY	Substitution	p.Asn30Ser
55	lymphoid	chr7	152163058	44	0.068	KMT2C	SG	Substitution	p.Arg3507X
56	lymphoid	chr7	152163364	68	0.074	KMT2C	SG	Substitution	p.Arg3405X
4	lymphoid	chr7	152185574	83	0.096	KMT2C	FS	Deletion	
79	lymphoid	chr12	49022763	57	0.088	KMT2D	NSY	Substitution	p.Arg5389Trp
32	lymphoid	chr12	49024887	64	0.047	KMT2D	SG	Substitution	p.Arg5282X
80	lymphoid	chr12	49032991	86	0.081	KMT2D	NSY	Substitution	p.Gln3905Leu
33	lymphoid	chr12	49051149	44	0.114	KMT2D	FS	Deletion	
609	myeloid	chr12	25225627	77	0.039	KRAS	NSY	Substitution	p.Ala146Val
608	myeloid	chr12	25225628	42	0.071	KRAS	NSY	Substitution	p.Ala146Thr
610	myeloid	chr12	25245348	63	0.048	KRAS	NSY	Substitution	p.Gly13Cys
611	myeloid	chr12	25245350	52	0.154	KRAS	NSY	Substitution	p.Gly12Val
104	lymphoid	chr2	140385944	39	0.154	LRP1B	NSY	Substitution	p.Arg3494Trp
105	lymphoid	chr2	140456528	55	0.109	LRP1B	NSY	Substitution	p.Thr3297Asn
153	lymphoid	chr8	97994561	31	0.194	MATN2	NSY	Substitution	p.Leu388Pro
154	lymphoid	chr8	98027730	69	0.072	MATN2	NSY	Substitution	p.Arg753Trp
64	lymphoid	chr1	148559835	75	0.093	NBPF14	NSY	Substitution	p.Ile1563Val
65	lymphoid	chr1	148559835	31	0.194	NBPF14	NSY	Substitution	p.Ile1563Val
37	lymphoid	chr17	16171996	22	0.273	NCOR1	ESS	Substitution	
837	myeloid	chr17	31201410	62	0.339	NF1	ESS	Substitution	
612	myeloid	chr17	31229201	52	0.288	NF1	FS	Insertion	
24	myeloid	chr17	31327527	103	0.039	NF1	SG	Substitution	p.Ser1766X
101	lymphoid	chr19	55954568	66	0.182	NLRP8	SG	Substitution	p.Trp170X
102	lymphoid	chr19	55954810	69	0.087	NLRP8	NSY	Substitution	p.Thr251Met
58	lymphoid	chr1	6554298	69	0.072	NOL9	NSY	Substitution	p.Val69Leu
5	lymphoid	chr1	119925561	58	0.052	NOTCH2	FS	Deletion	
6	lymphoid	chr1	119925561	56	0.054	NOTCH2	FS	Deletion	
7	lymphoid	chr1	119925561	69	0.072	NOTCH2	FS	Deletion	
177	myeloid	chr1	114716126	40	0.25	NRAS	NSY	Substitution	p.Gly12Asp
67	lymphoid	chr1	156873861	66	0.121	NTRK1	NSY	Substitution	p.Thr360Met
70	lymphoid	chr1	248203248	91	0.066	OR2M3	NSY	Substitution	p.Leu61Phe

83	lymphoid	chr13	25097645	73	0.068	PABPC3	NSY	Substitution	p.Thr483Ser
145	lymphoid	chr7	82760751	55	0.127	PCLO	NSY	Substitution	p.Ser5059Phe
146	lymphoid	chr7	82954382	72	0.194	PCLO	NSY	Substitution	p.Val2191Ile
147	lymphoid	chr7	82955074	62	0.081	PCLO	NSY	Substitution	p.Leu1960Ser
613	myeloid	chr6	78990981	34	0.118	PHIP	SG	Substitution	p.Gln736X
512	myeloid	chr6	79077869	53	0.132	PHIP	SG	Substitution	p.Gln29X
166	lymphoid	chrX	153776106	69	0.13	PLXNB3	NSY	Substitution	p.Arg1541Trp
167	lymphoid	chrX	153777313	72	0.153	PLXNB3	NSY	Substitution	p.Arg1678Gln
621	myeloid	chr17	60662996	25	0.16	PPM1D	SG	Substitution	p.Ser421X
637	myeloid	chr17	60663014	38	0.132	PPM1D	SG	Substitution	p.Trp427X
620	myeloid	chr17	60663015	49	0.082	PPM1D	SG	Substitution	p.Trp427X
228	myeloid	chr17	60663015	39	0.077	PPM1D	SG	Substitution	p.Trp427X
642	myeloid	chr17	60663125	61	0.197	PPM1D	FS	Deletion	
627	myeloid	chr17	60663128	55	0.127	PPM1D	FS	Deletion	
630	myeloid	chr17	60663137	71	0.07	PPM1D	SG	Substitution	p.Ser468X
643	myeloid	chr17	60663174	66	0.242	PPM1D	FS	Deletion	
619	myeloid	chr17	60663178	62	0.113	PPM1D	FS	Deletion	
625	myeloid	chr17	60663182	66	0.091	PPM1D	FS	Insertion	
631	myeloid	chr17	60663182	67	0.284	PPM1D	FS	Insertion	
636	myeloid	chr17	60663182	85	0.129	PPM1D	FS	Deletion	
641	myeloid	chr17	60663185	59	0.102	PPM1D	SG	Substitution	p.Leu484X
624	myeloid	chr17	60663262	62	0.21	PPM1D	FS	Deletion	
634	myeloid	chr17	60663262	83	0.036	PPM1D	SG	Substitution	p.Gln510X
644	myeloid	chr17	60663262	69	0.058	PPM1D	SG	Substitution	p.Gln510X
629	myeloid	chr17	60663269	78	0.064	PPM1D	FS	Deletion	
633	myeloid	chr17	60663269	73	0.205	PPM1D	FS	Deletion	
311	myeloid	chr17	60663273	74	0.189	PPM1D	FS	Insertion	
614	myeloid	chr17	60663281	48	0.229	PPM1D	SG	Substitution	p.Ser516X
617	myeloid	chr17	60663281	108	0.037	PPM1D	SG	Substitution	p.Ser516X
639	myeloid	chr17	60663304	51	0.059	PPM1D	SG	Substitution	p.Gln524X
645	myeloid	chr17	60663304	69	0.072	PPM1D	SG	Substitution	p.Gln524X

616	myeloid	chr17	60663333	65	0.046	PPM1D	FS	Deletion	
391	myeloid	chr17	60663333	69	0.072	PPM1D	FS	Deletion	
635	myeloid	chr17	60663352	51	0.098	PPM1D	SG	Substitution	p.Glu540X
640	myeloid	chr17	60663365	52	0.077	PPM1D	FS	Insertion	
615	myeloid	chr17	60663388	58	0.052	PPM1D	SG	Substitution	p.Arg552X
622	myeloid	chr17	60663388	79	0.076	PPM1D	SG	Substitution	p.Arg552X
626	myeloid	chr17	60663388	65	0.123	PPM1D	SG	Substitution	p.Arg552X
646	myeloid	chr17	60663388	66	0.045	PPM1D	SG	Substitution	p.Arg552X
623	myeloid	chr17	60663448	44	0.341	PPM1D	SG	Substitution	p.Arg572X
628	myeloid	chr17	60663448	48	0.062	PPM1D	SG	Substitution	p.Arg572X
632	myeloid	chr17	60663448	60	0.217	PPM1D	SG	Substitution	p.Arg572X
618	myeloid	chr17	60663475	48	0.188	PPM1D	SG	Substitution	p.Arg581X
638	myeloid	chr17	60663475	48	0.146	PPM1D	SG	Substitution	p.Arg581X
85	lymphoid	chr14	91481970	71	0.07	PPP4R3A	NSY	Substitution	p.Leu174Gln
92	lymphoid	chr16	23836239	68	0.103	PRKCB	NSY	Substitution	p.Arg22Cys
93	lymphoid	chr16	24092826	107	0.196	PRKCB	NSY	Substitution	p.Asn189Asp
601	myeloid	chr17	1659993	71	0.127	PRPF8	NSY	Substitution	p.Asp1598Glu
650	myeloid	chr17	1659994	38	0.184	PRPF8	NSY	Substitution	p.Asp1598Val
647	myeloid	chr17	1659995	45	0.067	PRPF8	NSY	Substitution	p.Asp1598Asn
649	myeloid	chr17	1659995	35	0.086	PRPF8	NSY	Substitution	p.Asp1598Asn
648	myeloid	chr17	1660436	34	0.206	PRPF8	NSY	Substitution	p.Cys1594Tyr
160	lymphoid	chr9	76706132	64	0.141	PRUNE2	FS	Deletion	
71	lymphoid	chr10	87957904	60	0.133	PTEN	NSY	Substitution	p.Ser229Leu
21	lymphoid	chr10	87960892	30	0.133	PTEN	ESS	Substitution	
22	lymphoid	chr10	87960892	22	0.182	PTEN	ESS	Substitution	
304	myeloid	chr12	112450353	68	0.324	PTPN11	NSY	Substitution	p.Asn58Ser
651	myeloid	chr12	112486532	51	0.059	PTPN11	NSY	Substitution	p.Val428Met
653	myeloid	chr12	112486532	57	0.07	PTPN11	NSY	Substitution	p.Val428Met
652	myeloid	chr12	112489105	65	0.046	PTPN11	NSY	Substitution	p.Gln510Pro
157	lymphoid	chr9	8376060	37	0.135	PTPRD	NSY	Substitution	p.Gln1513Lys
57	lymphoid	chr9	8521276	26	0.154	PTPRD	ESS	Substitution	

158	lymphoid	chr9	8524972	51	0.098	PTPRD	NSY	Substitution	p.Asn211Ser
654	myeloid	chr8	116852021	54	0.056	RAD21	SG	Substitution	p.Ser466X
655	myeloid	chr8	116861860	56	0.286	RAD21	SG	Substitution	p.Gln119X
84	lymphoid	chr13	48364952	52	0.192	RB1	NSY	Substitution	p.Thr307Ile
87	lymphoid	chr14	102592971	65	0.138	RCOR1	NSY	Substitution	p.Ala29Ser
88	lymphoid	chr14	102593077	60	0.167	RCOR1	NSY	Substitution	p.Ala64Val
43	lymphoid	chr6	117362803	89	0.18	ROS1	FS	Deletion	
148	lymphoid	chr8	10607568	107	0.121	RP1L1	NSY	Substitution	p.Leu2177Ser
149	lymphoid	chr8	10608500	29	0.172	RP1L1	NSY	Substitution	p.Glu1866Asp
98	lymphoid	chr19	1440048	98	0.061	RPS15	NSY	Substitution	p.Arg40Leu
99	lymphoid	chr19	1440173	53	0.132	RPS15	NSY	Substitution	p.Asp82His
100	lymphoid	chr19	1440173	37	0.162	RPS15	NSY	Substitution	p.Asp82His
657	myeloid	chr21	34834547	40	0.325	RUNX1	NSY	Substitution	p.Glu223Gly
656	myeloid	chr21	34886874	46	0.174	RUNX1	NSY	Substitution	p.Arg107His
114	lymphoid	chr4	25833002	35	0.2	SEL1L3	NSY	Substitution	p.Gly364Glu
82	lymphoid	chr12	121817206	57	0.105	SETD1B	NSY	Substitution	p.Lys963Asn
66	lymphoid	chr1	150961086	47	0.128	SETDB1	NSY	Substitution	p.Glu1009Asp
670	myeloid	chr2	197401818	58	0.138	SF3B1	NSY	Substitution	p.Tyr765Cys
662	myeloid	chr2	197401879	39	0.103	SF3B1	NSY	Substitution	p.Ala745Pro
617	myeloid	chr2	197402101	76	0.053	SF3B1	NSY	Substitution	p.Thr703Ala
147	myeloid	chr2	197402110	57	0.105	SF3B1	NSY	Substitution	p.Lys700Glu
658	myeloid	chr2	197402110	66	0.061	SF3B1	NSY	Substitution	p.Lys700Glu
659	myeloid	chr2	197402110	53	0.057	SF3B1	NSY	Substitution	p.Lys700Glu
557	myeloid	chr2	197402110	38	0.237	SF3B1	NSY	Substitution	p.Lys700Glu
558	myeloid	chr2	197402110	48	0.188	SF3B1	NSY	Substitution	p.Lys700Glu
668	myeloid	chr2	197402635	69	0.087	SF3B1	NSY	Substitution	p.Lys666Asn
673	myeloid	chr2	197402635	52	0.058	SF3B1	NSY	Substitution	p.Lys666Asn
676	myeloid	chr2	197402635	53	0.208	SF3B1	NSY	Substitution	p.Lys666Asn
660	myeloid	chr2	197402636	54	0.13	SF3B1	NSY	Substitution	p.Lys666Met
665	myeloid	chr2	197402636	53	0.132	SF3B1	NSY	Substitution	p.Lys666Thr
669	myeloid	chr2	197402636	49	0.102	SF3B1	NSY	Substitution	p.Lys666Arg

159	myeloid	chr2	197402636	43	0.419	SF3B1	NSY	Substitution	p.Lys666Thr
674	myeloid	chr2	197402636	55	0.327	SF3B1	NSY	Substitution	p.Lys666Thr
661	myeloid	chr2	197402637	54	0.056	SF3B1	NSY	Substitution	p.Lys666Glu
678	myeloid	chr2	197402645	43	0.163	SF3B1	NSY	Substitution	p.Thr663Ile
677	myeloid	chr2	197402647	72	0.097	SF3B1	NSY	Substitution	p.His662Gln
547	myeloid	chr2	197402678	69	0.043	SF3B1	NSY	Substitution	p.Cys652Tyr
663	myeloid	chr2	197402737	70	0.043	SF3B1	NSY	Substitution	p.Phe632Leu
666	myeloid	chr2	197402737	48	0.062	SF3B1	NSY	Substitution	p.Phe632Leu
672	myeloid	chr2	197402737	29	0.138	SF3B1	NSY	Substitution	p.Phe632Leu
663	myeloid	chr2	197402739	71	0.042	SF3B1	NSY	Substitution	p.Phe632Leu
666	myeloid	chr2	197402739	49	0.061	SF3B1	NSY	Substitution	p.Phe632Leu
672	myeloid	chr2	197402739	29	0.138	SF3B1	NSY	Substitution	p.Phe632Leu
663	myeloid	chr2	197402744	69	0.043	SF3B1	NSY	Substitution	p.Arg630Lys
666	myeloid	chr2	197402744	45	0.067	SF3B1	NSY	Substitution	p.Arg630Lys
671	myeloid	chr2	197402744	49	0.061	SF3B1	NSY	Substitution	p.Arg630Lys
672	myeloid	chr2	197402744	26	0.154	SF3B1	NSY	Substitution	p.Arg630Lys
667	myeloid	chr2	197402768	29	0.103	SF3B1	NSY	Substitution	p.Glu622Gly
675	myeloid	chr2	197402954	48	0.062	SF3B1	NSY	Substitution	p.Ala601Thr
664	myeloid	chr2	197402956	56	0.054	SF3B1	NSY	Substitution	p.Leu600Ser
8	lymphoid	chr1	15928254	41	0.122	SPEN	SG	Substitution	p.Arg672X
9	lymphoid	chr1	15929748	42	0.071	SPEN	SG	Substitution	p.Arg1170X
10	lymphoid	chr1	15929964	56	0.071	SPEN	SG	Substitution	p.Glu1242X
1	lymphoid	chr1	15935793	49	0.061	SPEN	SG	Substitution	p.Arg3185X
11	lymphoid	chr1	15935970	48	0.167	SPEN	FS	Insertion	
656	myeloid	chr17	76736877	63	0.222	SRSF2	NSY	Substitution	p.Pro95His
203	myeloid	chr17	76736877	54	0.204	SRSF2	NSY	Substitution	p.Pro95His
680	myeloid	chr17	76736877	90	0.267	SRSF2	NSY	Substitution	p.Pro95Leu
682	myeloid	chr17	76736877	48	0.229	SRSF2	NSY	Substitution	p.Pro95Leu
683	myeloid	chr17	76736877	107	0.112	SRSF2	NSY	Substitution	p.Pro95Leu
684	myeloid	chr17	76736877	55	0.218	SRSF2	NSY	Substitution	p.Pro95Leu
230	myeloid	chr17	76736877	75	0.16	SRSF2	NSY	Substitution	p.Pro95His

685	myeloid	chr17	76736877	80	0.112	SRSF2	NSY	Substitution	p.Pro95His
681	myeloid	chr17	76736878	62	0.339	SRSF2	NSY	Substitution	p.Pro95Thr
679	myeloid	chr17	76736991	59	0.322	SRSF2	NSY	Substitution	p.Phe57Tyr
445	myeloid	chr17	76736991	72	0.056	SRSF2	NSY	Substitution	p.Phe57Tyr
3	lymphoid	chr17	42322401	55	0.182	STAT3	NSY	Substitution	p.Asp661Val
38	lymphoid	chr17	42322402	66	0.045	STAT3	NSY	Substitution	p.Asp661Tyr
39	lymphoid	chr17	42322402	59	0.068	STAT3	NSY	Substitution	p.Asp661Tyr
81	lymphoid	chr12	57096862	30	0.2	STAT6	NSY	Substitution	p.Pro781Leu
141	lymphoid	chr6	152339298	35	0.2	SYNE1	NSY	Substitution	p.Asp4098Glu
142	lymphoid	chr6	152453692	47	0.149	SYNE1	NSY	Substitution	p.Val974Gly
163	lymphoid	chrX	71383149	47	0.106	TAF1	FS	Deletion	
164	lymphoid	chrX	71387378	71	0.197	TAF1	NSY	Substitution	p.Val782Met
165	lymphoid	chrX	71393340	65	0.077	TAF1	NSY	Substitution	p.Arg1031Cys
719	myeloid	chr4	105234173	78	0.115	TET2	FS	Deletion	
715	myeloid	chr4	105234264	57	0.053	TET2	SG	Substitution	p.Gln108X
787	myeloid	chr4	105234345	133	0.128	TET2	SG	Substitution	p.Glu135X
698	myeloid	chr4	105234354	62	0.113	TET2	SG	Substitution	p.Gln138X
725	myeloid	chr4	105234354	65	0.062	TET2	SG	Substitution	p.Gln138X
733	myeloid	chr4	105234354	87	0.034	TET2	SG	Substitution	p.Gln138X
621	myeloid	chr4	105234411	60	0.1	TET2	FS	Insertion	
202	myeloid	chr4	105234422	88	0.193	TET2	FS	Insertion	
690	myeloid	chr4	105234445	61	0.344	TET2	FS	Deletion	
689	myeloid	chr4	105234591	75	0.12	TET2	FS	Deletion	
750	myeloid	chr4	105234621	76	0.105	TET2	FS	Insertion	
713	myeloid	chr4	105234700	43	0.116	TET2	FS	Deletion	
745	myeloid	chr4	105234705	44	0.091	TET2	SG	Substitution	p.Gln255X
705	myeloid	chr4	105234763	57	0.07	TET2	FS	Deletion	
10	myeloid	chr4	105234763	54	0.148	TET2	FS	Deletion	
656	myeloid	chr4	105234885	53	0.321	TET2	FS	Deletion	
722	myeloid	chr4	105234903	47	0.213	TET2	SG	Substitution	p.Gln321X
734	myeloid	chr4	105235002	43	0.093	TET2	FS	Deletion	

780	myeloid	chr4	105235176	66	0.136	TET2	FS	Deletion	
770	myeloid	chr4	105235230	67	0.224	TET2	FS	Deletion	
753	myeloid	chr4	105235260	63	0.048	TET2	SG	Substitution	p.Gln440X
680	myeloid	chr4	105235323	57	0.088	TET2	SG	Substitution	p.Gln461X
687	myeloid	chr4	105235409	44	0.227	TET2	FS	Insertion	
706	myeloid	chr4	105235572	33	0.364	TET2	SG	Substitution	p.Arg544X
787	myeloid	chr4	105235611	74	0.459	TET2	SG	Substitution	p.Gln557X
39	myeloid	chr4	105235611	47	0.085	TET2	SG	Substitution	p.Gln557X
766	myeloid	chr4	105235619	67	0.149	TET2	SG	Substitution	p.Tyr559X
795	myeloid	chr4	105235633	58	0.276	TET2	SG	Substitution	p.Trp564X
688	myeloid	chr4	105235634	49	0.061	TET2	FS	Deletion	
386	myeloid	chr4	105235712	35	0.2	TET2	FS	Deletion	
757	myeloid	chr4	105235789	76	0.132	TET2	FS	Deletion	
781	myeloid	chr4	105235828	65	0.123	TET2	FS	Deletion	
774	myeloid	chr4	105235842	68	0.088	TET2	FS	Deletion	
735	myeloid	chr4	105235902	59	0.102	TET2	SG	Substitution	p.Gln654X
601	myeloid	chr4	105236016	49	0.102	TET2	FS	Insertion	
789	myeloid	chr4	105236024	71	0.183	TET2	FS	Insertion	
736	myeloid	chr4	105236071	76	0.145	TET2	FS	Deletion	
709	myeloid	chr4	105236127	80	0.075	TET2	SG	Substitution	p.Gln729X
740	myeloid	chr4	105236142	61	0.066	TET2	SG	Substitution	p.Gln734X
471	myeloid	chr4	105236149	73	0.233	TET2	FS	Insertion	
714	myeloid	chr4	105236166	71	0.211	TET2	SG	Substitution	p.Gln742X
798	myeloid	chr4	105236166	70	0.057	TET2	SG	Substitution	p.Gln742X
804	myeloid	chr4	105236215	63	0.127	TET2	FS	Deletion	
680	myeloid	chr4	105236227	71	0.113	TET2	FS	Insertion	
716	myeloid	chr4	105236310	72	0.069	TET2	FS	Deletion	
747	myeloid	chr4	105236422	66	0.106	TET2	FS	Insertion	
721	myeloid	chr4	105236433	109	0.046	TET2	SG	Substitution	p.Gln831X
723	myeloid	chr4	105236486	55	0.218	TET2	FS	Insertion	
318	myeloid	chr4	105236527	75	0.147	TET2	FS	Insertion	

691	myeloid	chr4	105236528	54	0.13	TET2	FS	Deletion	
741	myeloid	chr4	105236542	72	0.139	TET2	FS	Insertion	
355	myeloid	chr4	105236604	83	0.048	TET2	SG	Substitution	p.Gln888X
743	myeloid	chr4	105236679	74	0.108	TET2	FS	Deletion	
796	myeloid	chr4	105236685	96	0.062	TET2	FS	Deletion	
692	myeloid	chr4	105236688	56	0.161	TET2	SG	Substitution	p.Gln916X
718	myeloid	chr4	105236688	89	0.472	TET2	SG	Substitution	p.Gln916X
739	myeloid	chr4	105236688	82	0.037	TET2	SG	Substitution	p.Gln916X
758	myeloid	chr4	105236688	71	0.113	TET2	SG	Substitution	p.Gln916X
788	myeloid	chr4	105236688	83	0.048	TET2	SG	Substitution	p.Gln916X
806	myeloid	chr4	105236769	66	0.061	TET2	SG	Substitution	p.Gln943X
748	myeloid	chr4	105236804	52	0.058	TET2	SG	Substitution	p.Trp954X
797	myeloid	chr4	105236804	74	0.054	TET2	SG	Substitution	p.Trp954X
699	myeloid	chr4	105236831	99	0.273	TET2	FS	Deletion	
205	myeloid	chr4	105236838	96	0.083	TET2	SG	Substitution	p.Gln966X
755	myeloid	chr4	105236838	61	0.18	TET2	SG	Substitution	p.Gln966X
807	myeloid	chr4	105236838	81	0.062	TET2	SG	Substitution	p.Gln966X
695	myeloid	chr4	105236841	49	0.143	TET2	SG	Substitution	p.Gln967X
586	myeloid	chr4	105236841	94	0.064	TET2	SG	Substitution	p.Gln967X
760	myeloid	chr4	105236887	66	0.106	TET2	FS	Deletion	
707	myeloid	chr4	105236898	74	0.054	TET2	SG	Substitution	p.Gly986X
717	myeloid	chr4	105236941	62	0.306	TET2	FS	Deletion	
718	myeloid	chr4	105237030	50	0.32	TET2	SG	Substitution	p.Gln1030X
724	myeloid	chr4	105237034	57	0.281	TET2	FS	Deletion	
368	myeloid	chr4	105237096	58	0.121	TET2	SG	Substitution	p.Lys1052X
776	myeloid	chr4	105237281	54	0.13	TET2	FS	Insertion	
791	myeloid	chr4	105237289	65	0.154	TET2	FS	Insertion	
864	myeloid	chr4	105237352	64	0.047	TET2	ESS	Substitution	
865	myeloid	chr4	105237352	38	0.105	TET2	ESS	Substitution	
682	myeloid	chr4	105241344	29	0.241	TET2	FS	Deletion	
779	myeloid	chr4	105241360	59	0.169	TET2	NSY	Substitution	p.Glu1144Val

738	myeloid	chr4	105241411	93	0.129	TET2	NSY	Substitution	p.Arg1161Thr
800	myeloid	chr4	105241420	59	0.051	TET2	NSY	Substitution	p.Met1164Arg
731	myeloid	chr4	105242911	44	0.136	TET2	NSY	Substitution	p.Cys1193Tyr
793	myeloid	chr4	105242913	39	0.205	TET2	NSY	Substitution	p.Pro1194Ser
702	myeloid	chr4	105242922	31	0.161	TET2	NSY	Substitution	p.Lys1197Glu
697	myeloid	chr4	105242923	47	0.064	TET2	NSY	Substitution	p.Lys1197Arg
769	myeloid	chr4	105243604	48	0.083	TET2	NSY	Substitution	p.Leu1210Pro
756	myeloid	chr4	105243612	64	0.047	TET2	NSY	Substitution	p.Val1213Met
696	myeloid	chr4	105243615	39	0.487	TET2	NSY	Substitution	p.Arg1214Trp
540	myeloid	chr4	105243615	67	0.045	TET2	NSY	Substitution	p.Arg1214Trp
773	myeloid	chr4	105243621	48	0.062	TET2	SG	Substitution	p.Arg1216X
782	myeloid	chr4	105243621	54	0.056	TET2	SG	Substitution	p.Arg1216X
792	myeloid	chr4	105243622	70	0.3	TET2	NSY	Substitution	p.Arg1216Pro
732	myeloid	chr4	105243637	30	0.1	TET2	NSY	Substitution	p.Cys1221Tyr
799	myeloid	chr4	105243637	34	0.118	TET2	NSY	Substitution	p.Cys1221Tyr
803	myeloid	chr4	105243661	59	0.051	TET2	NSY	Substitution	p.Leu1229Arg
712	myeloid	chr4	105243709	49	0.082	TET2	NSY	Substitution	p.Tyr1245Cys
166	myeloid	chr4	105243756	39	0.103	TET2	NSY	Substitution	p.Arg1261Cys
772	myeloid	chr4	105243756	41	0.073	TET2	NSY	Substitution	p.Arg1261Cys
742	myeloid	chr4	105243759	24	0.125	TET2	NSY	Substitution	p.Arg1262Trp
45	myeloid	chr4	105259627	42	0.19	TET2	NSY	Substitution	p.Cys1271Ser
428	myeloid	chr4	105259633	44	0.136	TET2	NSY	Substitution	p.Cys1273Tyr
594	myeloid	chr4	105259638	53	0.094	TET2	NSY	Substitution	p.Gly1275Arg
684	myeloid	chr4	105259672	58	0.086	TET2	FS	Deletion	
805	myeloid	chr4	105259677	64	0.281	TET2	NSY	Substitution	p.Gly1288Arg
700	myeloid	chr4	105259681	78	0.141	TET2	NSY	Substitution	p.Cys1289Tyr
708	myeloid	chr4	105259689	80	0.075	TET2	FS	Deletion	
765	myeloid	chr4	105259691	96	0.031	TET2	NSY	Substitution	p.Ser1292Arg
560	myeloid	chr4	105259708	67	0.149	TET2	NSY	Substitution	p.Cys1298Tyr
866	myeloid	chr4	105259771	34	0.088	TET2	ESS	Substitution	
101	myeloid	chr4	105261757	41	0.195	TET2	ESS	Substitution	

729	myeloid	chr4	105261783	39	0.103	TET2	SG	Substitution	p.Gln1327X
462	myeloid	chr4	105261790	44	0.273	TET2	NSY	Substitution	p.Leu1329Pro
792	myeloid	chr4	105261846	65	0.169	TET2	SG	Substitution	p.Gln1348X
744	myeloid	chr4	105269629	38	0.158	TET2	NSY	Substitution	p.Ala1355Val
790	myeloid	chr4	105269640	39	0.128	TET2	NSY	Substitution	p.Arg1359Cys
777	myeloid	chr4	105269641	55	0.055	TET2	NSY	Substitution	p.Arg1359Leu
783	myeloid	chr4	105269641	41	0.171	TET2	NSY	Substitution	p.Arg1359His
805	myeloid	chr4	105269662	56	0.125	TET2	NSY	Substitution	p.Arg1366His
761	myeloid	chr4	105269674	71	0.211	TET2	NSY	Substitution	p.Gly1370Glu
763	myeloid	chr4	105269686	55	0.091	TET2	FS	Deletion	
786	myeloid	chr4	105269686	48	0.25	TET2	FS	Deletion	
275	myeloid	chr4	105269698	55	0.055	TET2	NSY	Substitution	p.Cys1378Tyr
767	myeloid	chr4	105269703	52	0.25	TET2	NSY	Substitution	p.His1380Tyr
681	myeloid	chr4	105269704	37	0.27	TET2	NSY	Substitution	p.His1380Pro
867	myeloid	chr4	105272563	37	0.405	TET2	ESS	Substitution	
754	myeloid	chr4	105272591	58	0.052	TET2	SG	Substitution	p.Arg1404X
703	myeloid	chr4	105272630	76	0.039	TET2	NSY	Substitution	p.Val1417Phe
707	myeloid	chr4	105272692	63	0.048	TET2	FS	Insertion	
302	myeloid	chr4	105272735	55	0.073	TET2	SG	Substitution	p.Arg1452X
794	myeloid	chr4	105272769	55	0.273	TET2	FS	Insertion	
778	myeloid	chr4	105272774	48	0.146	TET2	SG	Substitution	p.Arg1465X
227	myeloid	chr4	105272814	57	0.14	TET2	FS	Deletion	
762	myeloid	chr4	105272869	58	0.086	TET2	FS	Insertion	
727	myeloid	chr4	105272909	54	0.278	TET2	SG	Substitution	p.Gln1510X
730	myeloid	chr4	105275056	27	0.148	TET2	SG	Substitution	p.Arg1516X
494	myeloid	chr4	105275077	43	0.233	TET2	SG	Substitution	p.Gln1523X
720	myeloid	chr4	105275089	41	0.073	TET2	SG	Substitution	p.Gln1527X
118	myeloid	chr4	105275188	43	0.233	TET2	FS	Insertion	
726	myeloid	chr4	105275206	53	0.113	TET2	FS	Insertion	
686	myeloid	chr4	105275277	70	0.143	TET2	SG	Substitution	p.Tyr1589X
771	myeloid	chr4	105275317	44	0.227	TET2	SG	Substitution	p.Gln1603X

711	myeloid	chr4	105275328	60	0.133	TET2	FS	Deletion	
749	myeloid	chr4	105275349	56	0.161	TET2	FS	Deletion	
784	myeloid	chr4	105275589	35	0.229	TET2	SG	Substitution	p.Tyr1693X
471	myeloid	chr4	105275648	42	0.119	TET2	FS	Insertion	
806	myeloid	chr4	105275721	53	0.208	TET2	FS	Insertion	
752	myeloid	chr4	105275777	45	0.133	TET2	FS	Insertion	
764	myeloid	chr4	105275856	61	0.115	TET2	FS	Insertion	
684	myeloid	chr4	105275992	45	0.222	TET2	SG	Substitution	p.Gln1828X
802	myeloid	chr4	105276064	48	0.062	TET2	SG	Substitution	p.Gln1852X
728	myeloid	chr4	105276087	77	0.169	TET2	FS	Deletion	
768	myeloid	chr4	105276092	44	0.159	TET2	NSY	Substitution	p.Gly1861Glu
751	myeloid	chr4	105276098	88	0.034	TET2	NSY	Substitution	p.Ala1863Asp
694	myeloid	chr4	105276128	52	0.058	TET2	NSY	Substitution	p.Ile1873Thr
701	myeloid	chr4	105276128	70	0.1	TET2	NSY	Substitution	p.Ile1873Thr
704	myeloid	chr4	105276128	137	0.036	TET2	NSY	Substitution	p.Ile1873Thr
164	myeloid	chr4	105276128	58	0.121	TET2	NSY	Substitution	p.Ile1873Thr
758	myeloid	chr4	105276128	73	0.082	TET2	NSY	Substitution	p.Ile1873Thr
785	myeloid	chr4	105276128	56	0.054	TET2	NSY	Substitution	p.Ile1873Thr
330	myeloid	chr4	105276137	73	0.041	TET2	NSY	Substitution	p.Ala1876Val
737	myeloid	chr4	105276149	76	0.092	TET2	NSY	Substitution	p.Leu1880Pro
801	myeloid	chr4	105276152	33	0.091	TET2	NSY	Substitution	p.His1881Arg
693	myeloid	chr4	105276160	63	0.048	TET2	NSY	Substitution	p.Thr1884Ala
746	myeloid	chr4	105276190	46	0.065	TET2	NSY	Substitution	p.Pro1894Thr
775	myeloid	chr4	105276222	76	0.25	TET2	NSY	Substitution	p.His1904Gln
759	myeloid	chr4	105276248	71	0.042	TET2	NSY	Substitution	p.Gly1913Asp
222	myeloid	chr4	105276266	78	0.231	TET2	NSY	Substitution	p.Ala1919Asp
710	myeloid	chr4	105276301	85	0.118	TET2	SG	Substitution	p.Glu1931X
772	myeloid	chr4	105276395	51	0.412	TET2	NSY	Substitution	p.Pro1962Leu
51	lymphoid	chr6	137875748	63	0.048	TNFAIP3	SG	Substitution	p.Arg183X
52	lymphoid	chr6	137878913	48	0.062	TNFAIP3	SG	Substitution	p.Gln490X
811	myeloid	chr17	7673704	40	0.35	TP53	SG	Substitution	p.Arg306X

828	myeloid	chr17	7673779	65	0.046	TP53	NSY	Substitution	p.Asp281Tyr
829	myeloid	chr17	7673780	42	0.071	TP53	NSY	Substitution	p.Arg280Ser
813	myeloid	chr17	7673788	47	0.064	TP53	NSY	Substitution	p.Pro278Thr
827	myeloid	chr17	7673802	45	0.156	TP53	NSY	Substitution	p.Arg273His
831	myeloid	chr17	7673802	70	0.143	TP53	NSY	Substitution	p.Arg273His
805	myeloid	chr17	7673802	39	0.128	TP53	NSY	Substitution	p.Arg273Leu
810	myeloid	chr17	7674203	41	0.585	TP53	NSY	Substitution	p.Ile254Val
249	myeloid	chr17	7674203	28	0.429	TP53	NSY	Substitution	p.Ile254Val
809	myeloid	chr17	7674229	47	0.106	TP53	NSY	Substitution	p.Gly245Asp
824	myeloid	chr17	7674230	45	0.089	TP53	NSY	Substitution	p.Gly245Ser
819	myeloid	chr17	7674277	31	0.548	TP53	NSY	Substitution	p.Cys229Tyr
817	myeloid	chr17	7674952	46	0.065	TP53	NSY	Substitution	p.His193Gln
833	myeloid	chr17	7675076	32	0.094	TP53	NSY	Substitution	p.His179Arg
826	myeloid	chr17	7675088	42	0.071	TP53	NSY	Substitution	p.Arg175His
830	myeloid	chr17	7675089	54	0.111	TP53	NSY	Substitution	p.Arg175Gly
821	myeloid	chr17	7675092	43	0.093	TP53	NSY	Substitution	p.Arg174Gly
812	myeloid	chr17	7675119	49	0.061	TP53	SG	Substitution	p.Gln165X
825	myeloid	chr17	7675131	49	0.061	TP53	NSY	Substitution	p.Ala161Thr
832	myeloid	chr17	7675142	78	0.333	TP53	NSY	Substitution	p.Val157Gly
823	myeloid	chr17	7675190	69	0.043	TP53	NSY	Substitution	p.Cys141Tyr
227	myeloid	chr17	7676040	41	0.22	TP53	NSY	Substitution	p.Arg110Pro
820	myeloid	chr17	7676064	48	0.333	TP53	NSY	Substitution	p.Thr102Asn
808	myeloid	chr17	7676148	70	0.043	TP53	NSY	Substitution	p.Ala74Val
815	myeloid	chr17	7676387	49	0.469	TP53	NSY	Substitution	p.Val31Ile
816	myeloid	chr17	7676387	98	0.082	TP53	NSY	Substitution	p.Val31Ile
822	myeloid	chr17	7676387	32	0.5	TP53	NSY	Substitution	p.Val31Ile
818	myeloid	chr17	7676564	39	0.359	TP53	NSY	Substitution	p.Glu11Gln
704	myeloid	chr17	7676580	78	0.038	TP53	NSY	Substitution	p.Gln5His
814	myeloid	chr17	7676584	35	0.086	TP53	NSY	Substitution	p.Pro4Leu
89	lymphoid	chr14	102876517	33	0.182	TRAF3	NSY	Substitution	p.Ala188Ser
90	lymphoid	chr14	102905741	53	0.113	TRAF3	NSY	Substitution	p.Phe555Ser

63	lymphoid	chr1	52435451	27	0.185	TUT4	NSY	Substitution	p.Arg1393Cys
155	lymphoid	chr8	102277120	75	0.107	UBR5	FS	Insertion	
156	lymphoid	chr8	102329172	44	0.136	UBR5	NSY	Substitution	p.Thr415Ile
94	lymphoid	chr17	5133485	30	0.2	USP6	NSY	Substitution	p.Gly107Cys
835	myeloid	chr11	32417618	56	0.054	WT1	SG	Substitution	p.Cys308X
834	myeloid	chr11	32428030	40	0.1	WT1	FS	Deletion	
86	lymphoid	chr14	100239638	65	0.123	YY1	NSY	Substitution	p.Leu132Ile
113	lymphoid	chr3	101651425	88	0.057	ZBTB11	FS	Deletion	
62	lymphoid	chr1	37482878	88	0.125	ZC3H12A	NSY	Substitution	p.Arg356Gln
109	lymphoid	chr20	53582403	52	0.154	ZNF217	NSY	Substitution	p.Asp142Tyr
139	lymphoid	chr6	87259999	92	0.054	ZNF292	FS	Deletion	
140	lymphoid	chr6	87261310	34	0.176	ZNF292	NSY	Substitution	p.Val2561Met

Supplemental Table 4: List of incident hematological malignancies in 10,067 individuals overall, and by CHIP/MBL status

Lymphoid Type (N=94)	Overall	No MBL/CHIP	CHIP only	MBL only	MBL/CHIP
Acute Lymphoblastic Leukemia (ALL)	1 (1.1%)		1		
Chronic Lymphocytic Leukemia	16 (17.1%)	1	2	12	1
Cutaneous Marginal B-Cell Lymphoma	1 (1.1%)			1	
Hodgkin Lymphoma	1 (1.1%)	1			
Large Granular Lymphocytic Leukemia NK-cell	1 (1.1%)	1			
Large Granular Lymphocytic Leukemia T-cell	1 (1.1%)				1
Multiple Myeloma	17 (18.1%)	11	3	2	1
Non-Hodgkin Lymphoma, not otherwise specified	2 (2.1%)	2			
Cutaneous T-Cell Lymphoma	4 (4.3%)	4			
Diffuse large B-cell lymphoma	16 (17.0%)	11	1	3	1
Follicular Lymphoma	14 (14.9%)	7	1	5	1
Lymphoplasmacytic	2 (2.1%)	2			
Mantle Cell	6 (6.4%)	2		2	2
Marginal Zone	3 (3.2%)	1		2	
Marginal Zone-Extra nodal	2 (2.1%)	1		1	
Marginal Zone-MALT	1 (1.1%)				1
Marginal Zone-Splenic	1 (1.1%)			1	
Plasmacytoma Nos	2 (2.1%)	2			
Polymorphic PTLD	1 (1.1%)	1			
Solitary Extramedullary Plasmacytoma	1 (1.1%)	1			
T-Cell Lymphoma	1 (1.1%)	1			

Myeloid Type (N=46)	Overall	No MBL/CHIP	CHIP only	MBL only	MBL/CHIP
Acute Myeloid Leukemia (AML)	12 (26.1%)	7	2	3	
Chronic Myeloid Leukemia (CML)	1 (2.2%)			1	
Chronic Myelomonocytic Leukemia (CMML)	3 (6.5%)		3		

Essential Thrombocythemia	4 (8.7%)	2	1		1
Myelodysplastic syndrome (MDS)	12 (26.1%)	6	3	2	1
Myeloproliferative Disease (MPD)	5 (10.9%)	1	2	1	1
Mixed Phenotype Acute Leukemia, T/myeloid	1 (2.2%)		1		
Polycythemia	8 (17.4%)	5	2		1

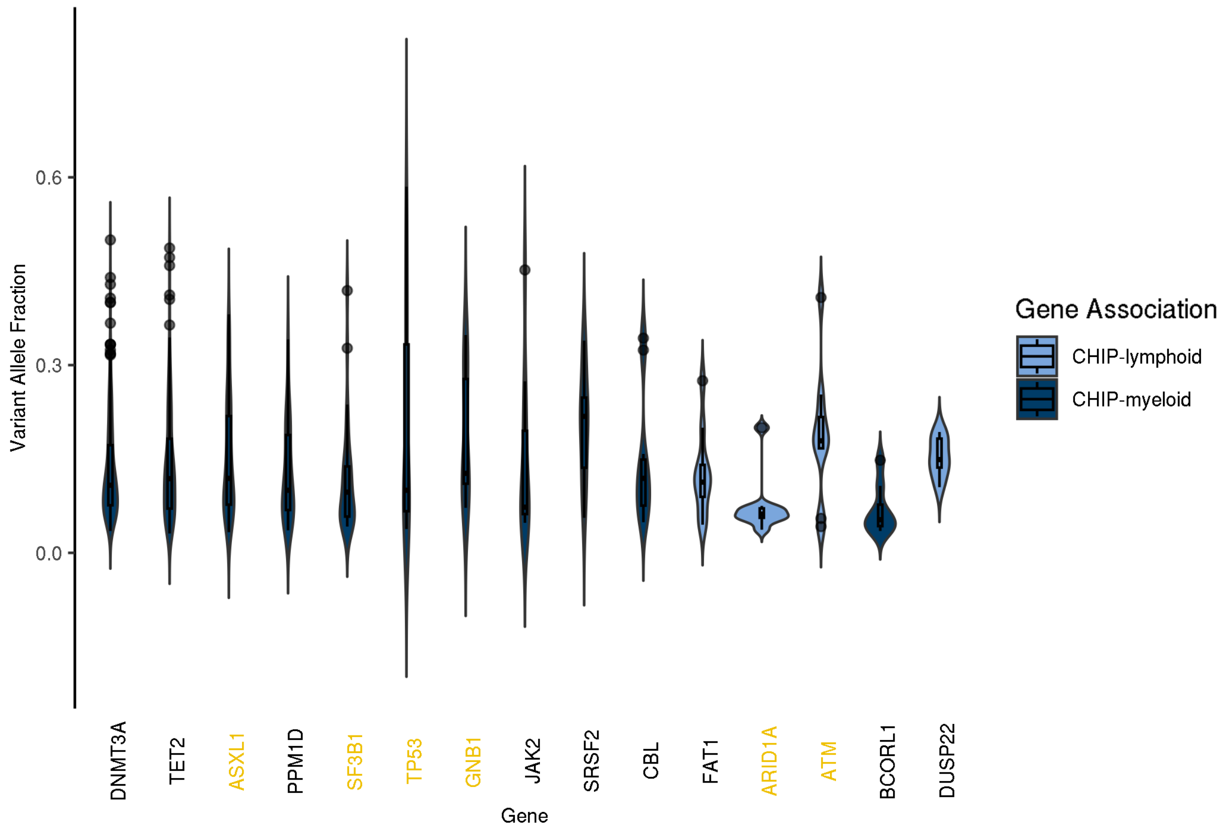

Supplemental Figure 1) Distribution of variant allele fraction (VAF) across the top 15 genes with CHIP variants. Gene names are colored (gold) to reflect the subset of CHIP genes association with chronic lymphocytic leukemia (CLL). Variants with VAF =>50% were variants in known hot spots or observed in multiple participants with a range of VAFs (indicative of a somatic variant). For example, the *DNMT3A* variant, p.Arg320X, was observed four times in our cohort with VAFs ranging from 0.042 - 0.50.

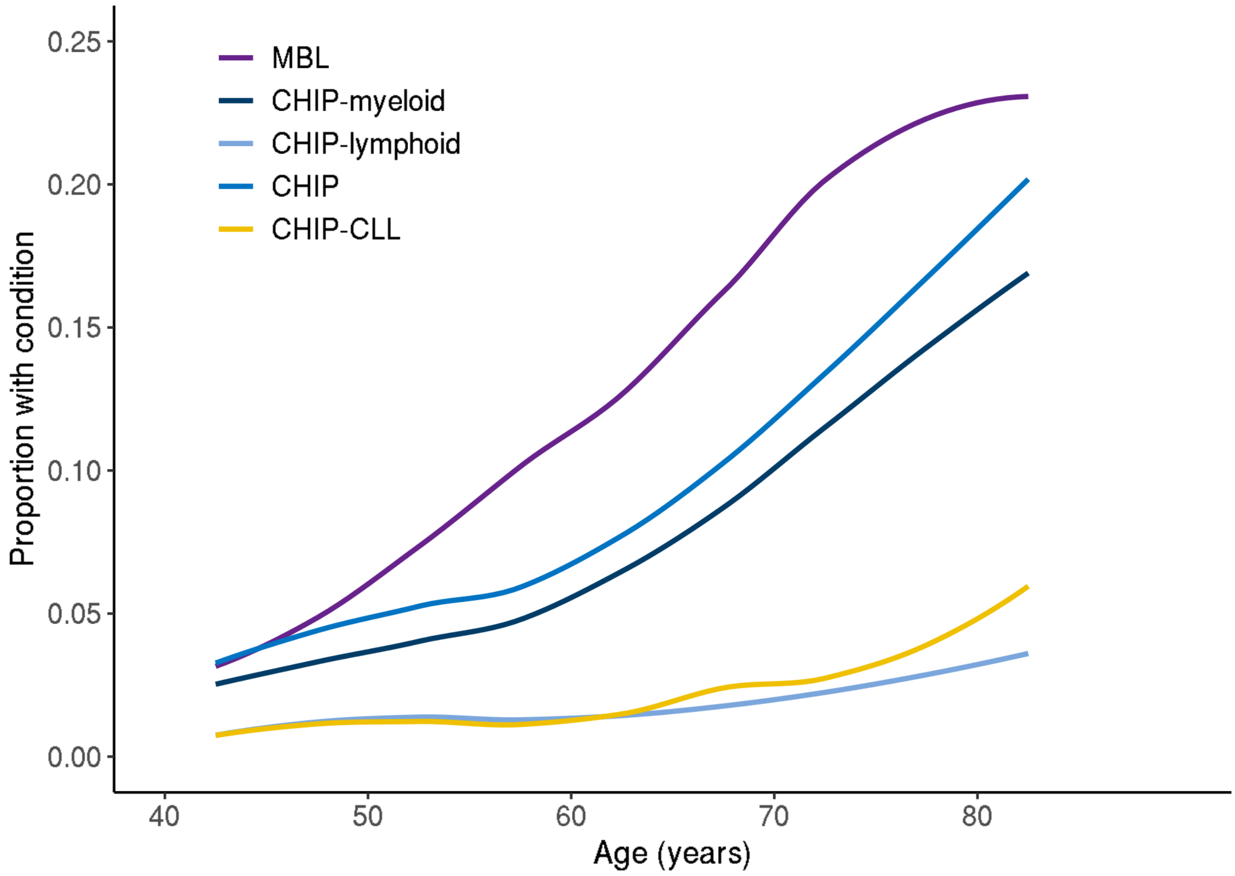

Supplemental Figure 2) Prevalence of each precursor conditions across age. Clonal hematopoiesis of indeterminate potential (CHIP) is defined as variants in genes associated with either myeloid (N=56 genes) or lymphoid (N=235 genes). CHIP-myeloid is defined as variants in 56 genes associated with myeloid malignancy. CHIP-lymphoid is defined as variants in 235 genes associated with lymphoid malignancy. CHIP-CLL is defined as variants in a subset of genes associated with chronic lymphocytic leukemia (CLL). CHIP-myeloid and CHIP-lymphoid are mutually exclusive, but CHIP-CLL includes genes from both CHIP-myeloid and CHIP-lymphoid.

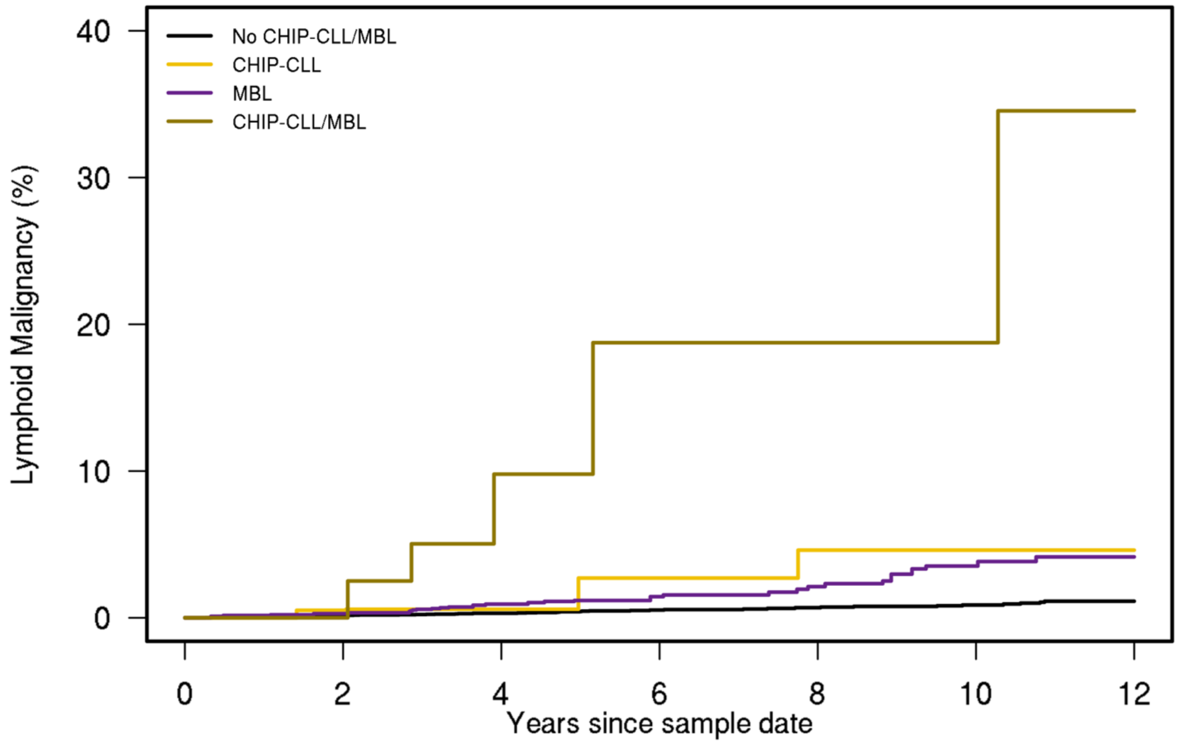

No CHIP-CLL/MBL	8396	7794	6082	3193	2154	1664	1152
CHIP-CLL	157	141	118	64	43	27	13
MBL	1469	1351	1055	461	270	200	126
CHIP-CLL/MBL	45	40	22	10	5	4	1

Variable	Total	Event	HR (95% CI)	P-value
MBL	1469	32	3.31 (2.10, 5.20)	<0.001
CHIP-CLL	157	4	3.62 (1.30, 10.06)	0.0135
CHIP-CLL/MBL	45	5	20.93 (8.24, 53.19)	<0.001

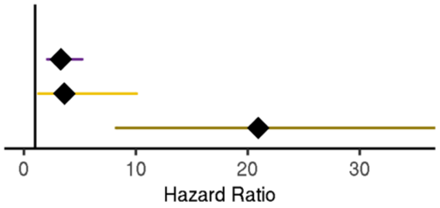

Supplemental Figure 3) Cumulative incident lymphoid malignancy by precursor condition groups. CHIP-CLL is defined as variants in a subset of 43 genes associated with chronic lymphocytic leukemia (CLL).
