# Supplementary material for: CHIP amplifies the risk of lymphoid malignancies in individuals with monoclonal B-cell lymphocytosis (MBL)
Source: Blood Cancer J. 2025 Nov 6;15(1):195. doi: 10.1038/s41408-025-01385-8 (PMC12592468; doi:10.1038/s41408-025-01385-8)
Supplement: Supplementary file 2 — Supplementary figure legend [file 41408_2025_1385_MOESM2_ESM.docx]

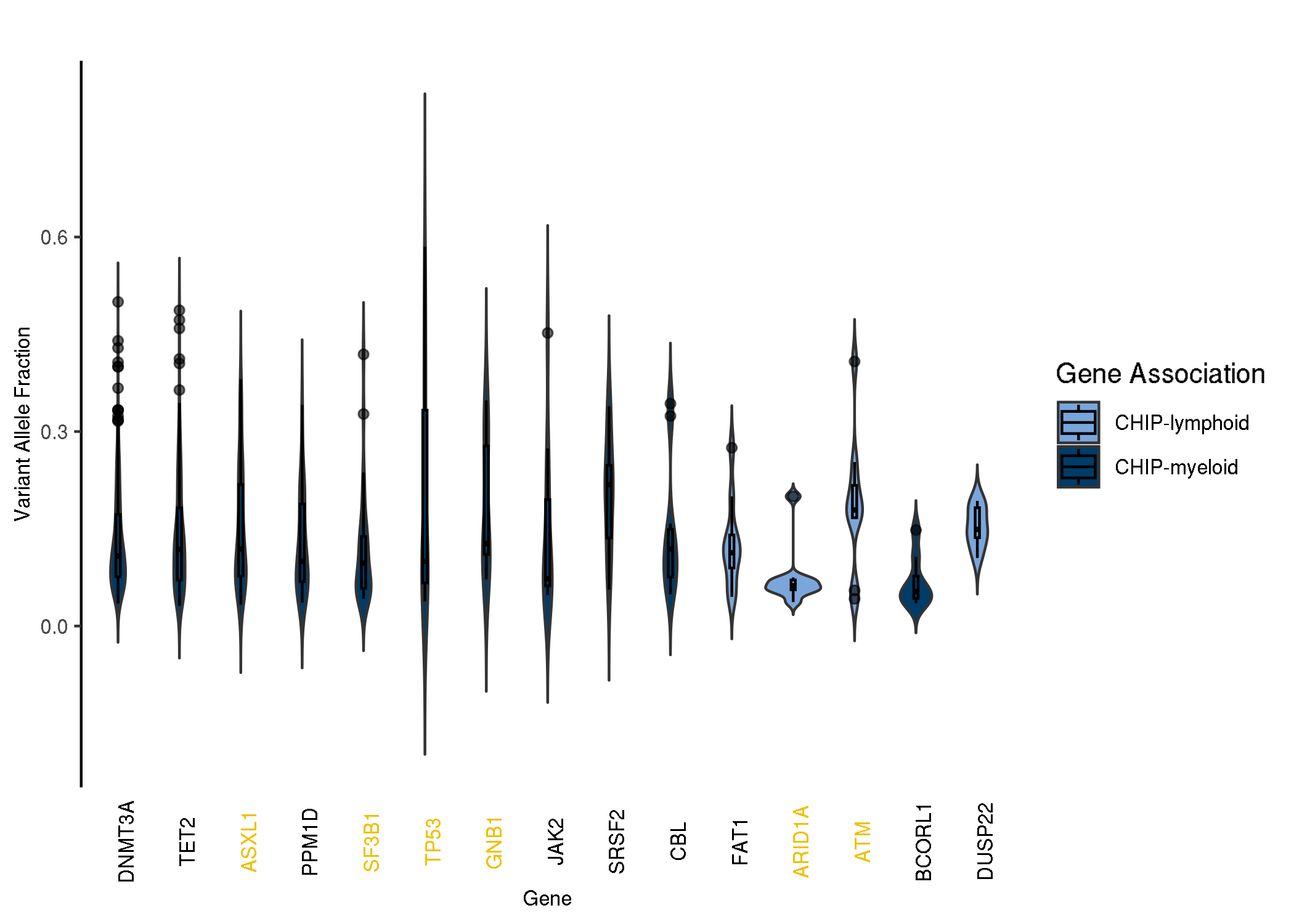


**Supplemental Figure 1)** Distribution of variant allele fraction across the top 15 genes with CHIP variants. Gene names are colored (gold) to reflect the subset of CHIP genes association with chronic lymphocytic leukemia (CLL).


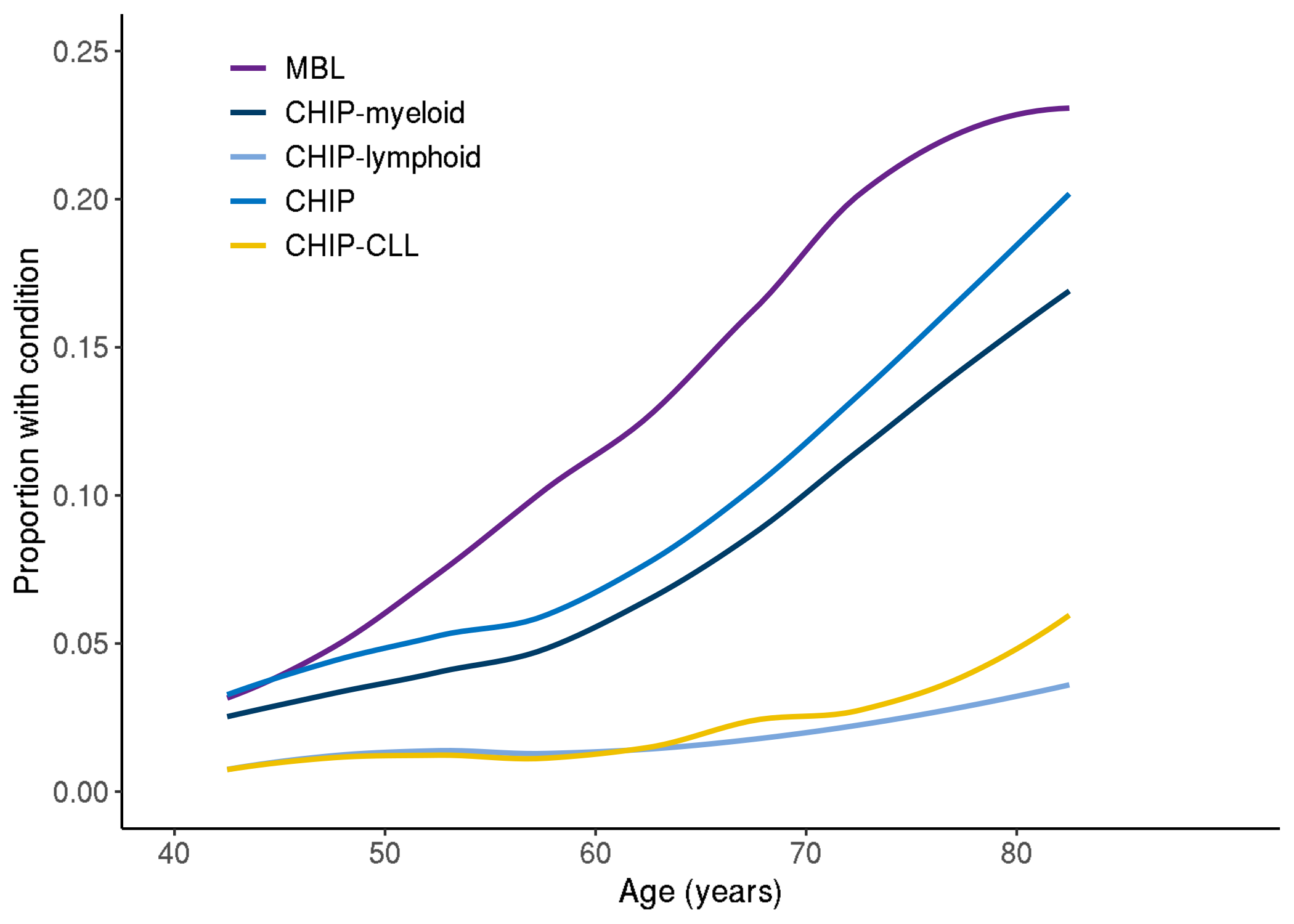


**Supplemental Figure 2)** Prevalence of precursor conditions across age. Clonal hematopoiesis of indeterminate potential (CHIP) is defined as variants in genes associated with either myeloid (N=56 genes) or lymphoid (N=235 genes). CHIP-myeloid is defined as variants in 56 genes associated with myeloid malignancy. CHIP-lymphoid is defined as variants in 235 genes associated with lymphoid malignancy. CHIP-CLL is defined as variants in a subset of genes associated with chronic lymphocytic leukemia (CLL). CHIP-myeloid and CHIP-lymphoid are mutually exclusive, but CHIP-CLL includes genes from both CHIP-myeloid and CHIP-lymphoid.


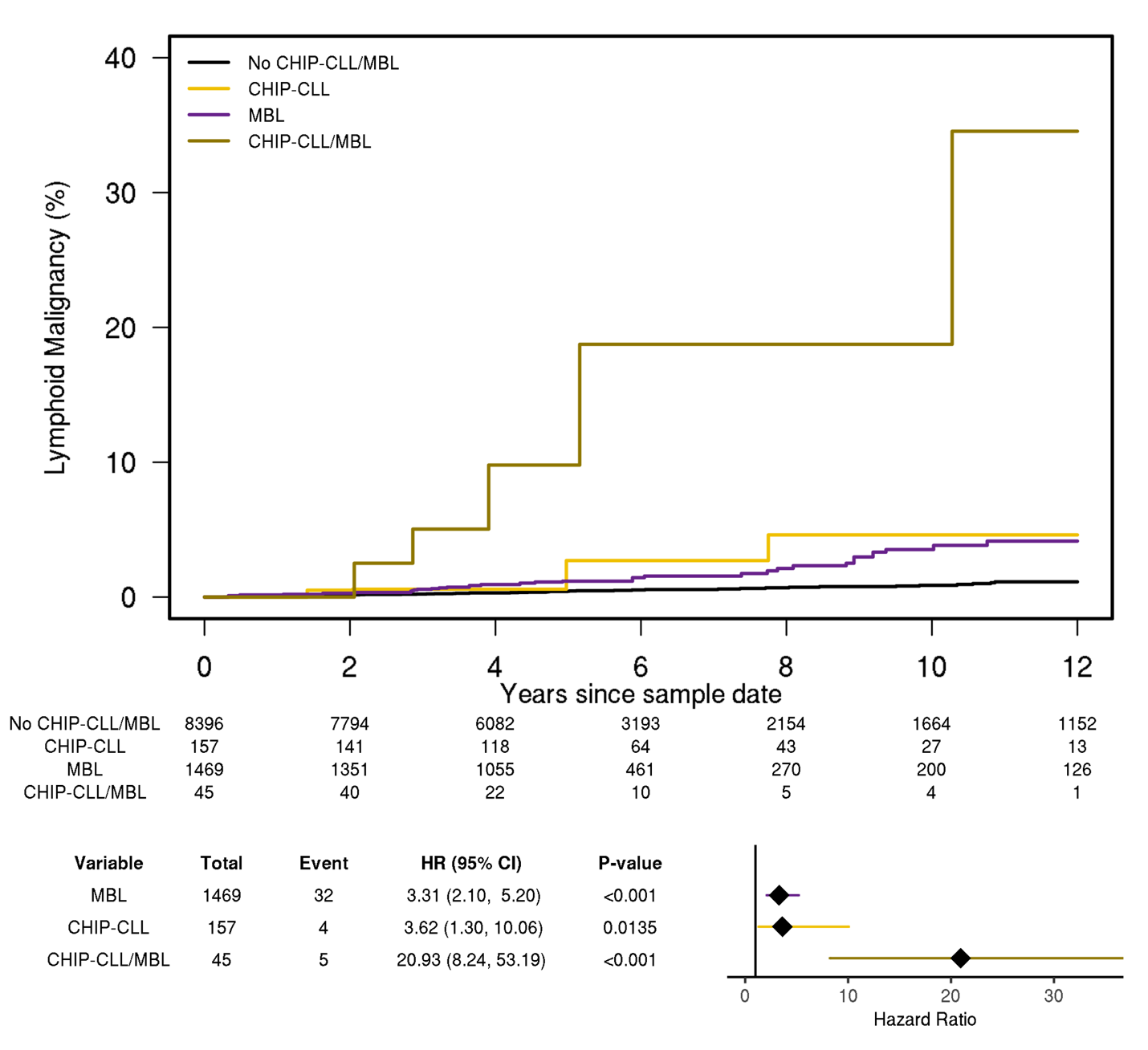


**Supplemental Figure 3)** Incident lymphoid malignancy by precursor condition groups. CHIP-CLL is defined as variants in a subset of 43 genes associated with chronic lymphocytic leukemia (CLL).


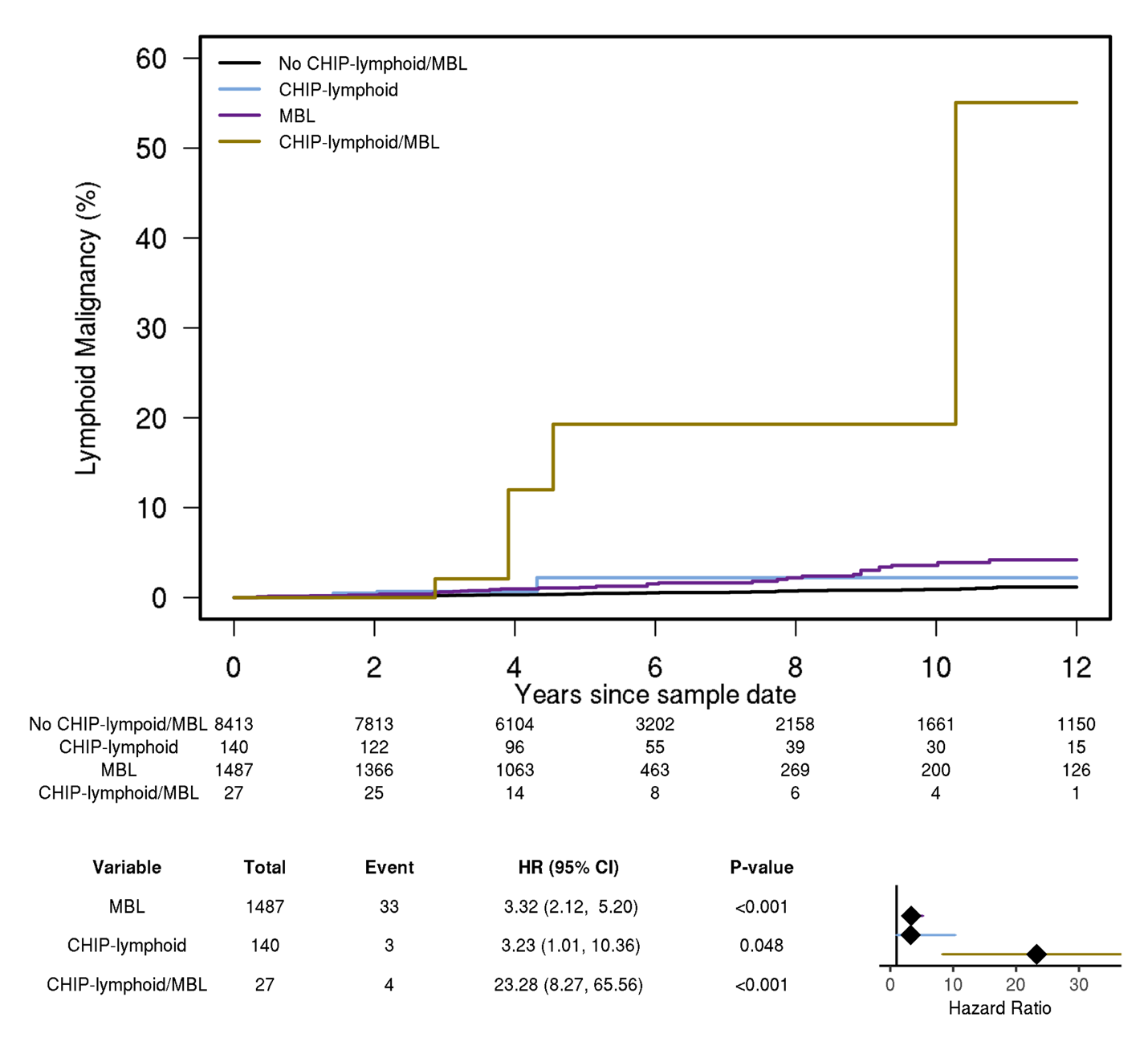


**Supplemental Figure 4)** Incident lymphoid malignancy by precursor condition groups. CHIP-lymphoid is defined as variants in 235 genes associated with lymphoid malignancy.
